# MiR-612 regulates invadopodia of hepatocellular carcinoma by HADHA-mediated lipid reprogramming

**DOI:** 10.1186/s13045-019-0841-3

**Published:** 2020-02-07

**Authors:** Yang Liu, Li-Li Lu, Duo Wen, Dong-Li Liu, Li-Li Dong, Dong-Mei Gao, Xin-Yu Bian, Jian Zhou, Jia Fan, Wei-Zhong Wu

**Affiliations:** 1 Liver Cancer Institute, Zhongshan Hospital, Key Laboratory of Carcinogenesis and Cancer Invasion, Ministry of Education, Fudan University, 180 Fenglin Road, Shanghai, 200032 China; 2 Department of Oral Maxillofacial-Head and Neck Oncology, Shanghai Ninth People’s Hospital, School of Medicine, Shanghai Jiao Tong University, Shanghai, China; 3 Department of Head and Neck Surgery, Fudan University Shanghai Cancer Center, Shanghai, 200032 China; 4 Department of Radiation Oncology, Shanghai General Hospital, Shanghai Jiaotong University, Shanghai, 200080 China; 5 Department of Liver Surgery and Transplantation, Zhongshan Hospital, Fudan University, Shanghai, 200032 China

**Keywords:** miR-612, Hepatocellular carcinoma, Invadopodia, β-Oxidation, Metastasis

## Abstract

**Background:**

MicroRNA-612 (miR-612) has been proven to suppress EMT, stemness, and tumor metastasis of hepatocellular carcinoma (HCC) via PI3K/AKT2 and Sp1/Nanog signaling. However, its biological roles on HCC progression are far from elucidated.

**Methods:**

We found direct downstream target of miR-612, *hadha* by RNA immunoprecipitation and sequencing. To explore its biological characteristic, potential molecular mechanism, and clinical relevance in HCC patients, we performed several in-vitro and in-vivo models, as well as human tissue chip.

**Results:**

Ectopic expression of miR-612 could partially reverse the level of HADHA, then suppress function of pseudopods, and diminish metastatic and invasive potential of HCC by lipid reprogramming. In detail, miR-612 might reduce invadopodia formation via HADHA-mediated cell membrane cholesterol alteration and accompanied with the inhibition of Wnt/β-catenin regulated EMT occurrence. Our results showed that the maximum oxygen consumption rates (OCR) of HCCLM3^miR-612-OE^ and HCCLM3^*hadha*-KD^ cells were decreased nearly by 40% and 60% of their counterparts (*p* < 0.05). The levels of acetyl CoA were significantly decreased, about 1/3 (*p* > 0.05) or 1/2 (*p* < 0.05) of their controls, in exogenous miR-612 or *hadha*-shRNA transfected HCCLM3 cell lines. Besides, overexpression of hadha cell lines had a high expression level of total cholesterol, especially 27-hydroxycholesterol (*p* < 0.005). SREBP2 protein expression level as well as its downstream targets, HMGCS1, HMGCR, MVD, SQLE were all deregulated by HADHA. Meanwhile, the ATP levels were reduced to 1/2 and 1/4 in HCCLM3^miR-612-OE^ (*p* < 0.05) and HCCLM3^*hadha*-KD^ (*p* < 0.01) respectively. Moreover, patients with low miR-612 levels and high HADHA levels had a poor prognosis with shorter overall survival.

**Conclusion:**

miR-612 can suppress the formation of invadopodia, EMT, and HCC metastasis and by HADHA-mediated lipid programming, which may provide a new insight of miR-612 on tumor metastasis and progression.

## Introduction

Hepatocellular carcinoma (HCC) is an aggressive cancer with poor long-term survival. Every year, up to 600,000 people die from the disease in the worldwide, and it has been a serious issue to human health [1, 2]. High recurrence and metastasis rate of HCC after surgical resection is still a Gordian knot to further improve the patients’ survival. Therefore, the exploration of underlying mechanism of HCC recurrence and metastasis has been an important clinical significance for early diagnosis and intervention.

Normally, cell morphology and function are elaborately orchestrated by dynamic metabolisms of carbohydrates, lipids, and amino acids. Dysfunctions of metabolic reprogramming have been found to be an important characteristic of tumor cells during tumorigenesis and metastasis. Even in normoxia, tumor cells are inclined to produce ATP by glycolysis, which is called as aerobic glycolysis or Warburg effects [3]. Although less efficient than oxidative phosphorylation, tumor cells usually turnover glucoses quickly in glycolytic pathway to meet their aggressive needs on energy and produce an intermediate, glutamine, the fastest-consuming amino acid in tumor microenvironment [4]. The latter may provide carbon and nitrogen sources for de novo syntheses of amino acids, nucleotides, and lipids. Recently, many evidences suggest that the abnormities of lipid- and cholesterol-metabolisms are also involved in tumorigenesis and progression. Lipids are reported to be another important energy source to fuel tumor metastasis [5]. Lipids, together with cholesterol, could form lipid rafts on cell membrane where a lot of receptors, ligands, and iron-channel proteins are aggregated as a functional unit. Destruction of lipid rafts is enough to prevent cell proliferation and tumor growth [6]. In addition, invadopodia, another prominent membranous structure in migrated and invasive cancer cells, is closely regulated by lipid- and cholesterol-metabolisms. The cholesterol-biosynthesis pathway is under tight regulation by transcription factors, such as sterol regulatory element-binding protein 1 and 2 (SREBP1 and 2). The accumulation of SREBP2 in nucleus binds to sterol response elements (SREs) to activate the expression of cholesterol-biosynthesis enzymes, such as 3-hydroxy-3-methylglutaryl-CoA reductase (HMGCR) and squalene epoxidase (SQLE) [7]. Whether this cascade could regulate cholesterol metabolism and invadopodia formation of HCC cells were still unknown. In virtue of invadopodia, cancer cells can degrade extracellular matrix, penetrate through blood vessels, and finally disseminate into distant target organs [8]. Due to its important roles in tumor metastasis, a lot of components and regulators of invadopodia, such as integrins, actin-binding proteins, scaffolders [9, 10], as well as signaling adaptors, are widely studied [11]. However, the roles of lipid metabolisms on the formation and the function of invadopodia in HCC still need to be unveiled.

Non-coding RNAs (ncRNAs) have emerged as one important kind of molecules that can regulate altered genes contributing, to the establishment of metabolic reprogramming [12]. For instance, SNHG16 could be regulated by the Wnt pathway in colorectal cancer and affect genes involved in lipid metabolism [13]. HULC functions as an oncogene in hepatoma cells, acting mechanistically by deregulating lipid metabolism through a signaling pathway involving miR-9, PPARA, and ACSL1 [14]. MicroRNAs (miRNAs), as a class of endogenous small noncoding RNAs, can regulate target protein expression at posttranscriptional level [15, 16]. To date, multiple miRNAs have been discovered to involve in the regulation of lipid metabolism, such as miR-30c, miR-122, etc. [17, 18]. In our previous study, miR-612 was found to have robustly suppressive effects on HCC proliferation and metastasis [19]. And 167 genes were predicted as potential targets of miR-612 by miRanda, TargetScan, and miRTarget2. Two pivotal genes were drawn out from these predicted genes in gene interaction network based on function enrichment analysis (Additional file 1: Figure S1A). One is *akt2*, which has been confirmed to mediate the suppressive effects of miR-612 in HCC invasive-metastatic cascade in our previously study. The other is *hadha*, whose roles in HCC metastasis are unknown by far. As *hadha* encodes the alpha subunit of mitochondrial trifunctional protein, hydroxyacyl-CoA dehydrogenase/3-ketoacyl-CoA thiolase/enoyl-CoA hydratase, the enzyme complex catalyze the three steps of beta-oxidation of fatty acids in mitochondria: long-chain 3-hydroxyacyl-CoA dehydrogenase (LCHAD), long-chain enoyl-CoA hydratase, and long-chain thiolase activities [20]. Therefore, we supposed that miR-612 might regulate tumor invasiveness and metastasis by HADHA-mediated lipid reprogramming and probably resulting in the abnormity of invadopodia in structure and function in HCC.

In this study, we mainly focused the roles of miR-612 on lipid reprogramming, invasiveness, and metastasis of HCC, which may lay a basis for its clinical application in precision diagnosis and therapy of metastatic HCC.

## Materials and methods

### Cell lines and cell culture

Human HCC cell line, HCCLM3, was established at the Liver Cancer Institute, Zhongshan Hospital, Fudan University, Shanghai, China [21, 22]. HepG2 cell line was purchased from the Shanghai Cell Bank, Chinese Academy of Sciences (CAS). HCCLM3 cells express low endogenous levels of miR-612 with relatively high metastatic potentials, whereas HepG2 cells express high endogenous levels of miR-612 with low metastatic potentials [19, 23, 24]. We transiently transfected with miR-612 mimic oligonucleotides (mimics) or inhibitory oligonucleotides (inhibitors) into HCCLM3 or HepG2 cells. hU6-MCS-CMV-shRNA-eGFP and Ubi-MCS-HADHA-3FLAG-SV40-Cherry lentiviral vectors were purchased from Genomeditech (Shanghai, China). They were transfected into HCCLM3 or HepG2 cells, respectively. The target sequences of si-HADHA was TGGTGACAAGATTTGTGAA, and HADHA-cDNA primer sequences were listed as follows: Forward: GAGGATCCCCGGGTACCGGTCGCCACCATGGCGGAGCCGAGCGGC; Reward: TCACCATGGTGGCGACCGGGCTGACACTCAACTGAGCA. All negative control cells either with mocked oligonucleotides or mocked-shRNA or mocked-cDNA lentiviral vectors were named as HCCLM3^NC^ and HepG2^NC^. Wild-type (WT) cell lines were with no treatments. All these cells were cultured under standard conditions, DMEM (GE, Utah, USA) supplemented with 10% FBS (GE, Utah, USA), penicillin (100 IU/ml), and streptomycin sulfate (100 μg/ml) and routinely maintained in a humidified incubator at 5% CO_2_ at 37 °C.

### Gelatin invadopodia assay

QCM™ gelatin invadopodia assay (No.ECM671, Millipore, MA, USA) was used for rapid detection of matrix degradation [25]. In belief, 2 × 10^4^/500 μl of HCC cells were added into glass dish pre-prepared with fluorescent substrate. Then the cells were cultured in the dark at room temperature for 15–30 min and sequentially in an incubator for 48 h. After fixed with 3.7% formaldehyde for 30 min, and stained with FITC-phalloidin and DAPI in blocking/permeabilization buffer for 1 h respectively, the cells were removed from staining solution and rinsed twice with fluorescent staining buffer using 500 μl/well. Laser scanning confocal microscope-TCS SP5 (Leica) was used to capture images.

### β-oxidation activity detection

HCC cells were cultured in XF 96-well microplate. The medium was replaced 24 h before the experiment and the cells were washed twice with the FAO assay medium 45 min before the assay. Fifteen microliter per well 40 mM Eto was added in a column of cells as positive control group and incubated for 15 min at 37 °C in a CO_2_ free incubator. One hundred thirty five microliter per well of FAO assay medium was added to the cells and continued to incubate for 30–45 min. The XF cell culture microplate was inserted into the XF96 analyzer to detect. 2.5 μg/ml oligomycin (oligo), 1.6 μM FCCP, and 2 μM Rotenone/4 μM Antimycin A (Rtn/AA) were added to the cell cultures at 22 min, 38 min, and 52 min in order.

### ATP assay

ATP level in sample was tested by a commercial kit (No. A095, Jiancheng Bioengineering Institute, Nanjing, China). In brief, 10^6^ of cell pellets were re-suspended in 500 μl ddH_2_O. After homogenized and boiled at 90–100 °C for 10 min, the mixture was vortexed and centrifuged. The supernatant was collected to set up a reaction system as described in Additional file 5: Table S3. The OD value of each sample was measured by spectrophotometer (λ = 636 nm). The ATP level (μmol/g) = (OD_measured_ − OD_control_) / (OD_standard_ − OD_blank_) × standard concentration (1 × 10^3^ μmol/L) × sample before dilution / sample protein concentration (g/l).

### Cholesterol quantitation assay

The concentration of cholesterol was measured by Cholesterol/Cholesteryl Ester Quantitation Assay kit (No. ab65359, Abcam, MA, USA). In belief, 1 × 10^6^ HCC cells with indicated treatment were centrifuged and the pellet was re-suspended in 200 μl of Chloroform:Isopropanol:NP-40 (7:11:0.1) solution using a microhomogenizer. Then the liquid in organic phase was taken out and transferred into a new tube after spinned at 15,000 g for 10 min, air dried at 50 °C, and vacuumed for 30 min. The dried lipids were re-dissolved in 200 μl kit provided Assay Buffer by virtue of sonicator and vortex mixer, and set up a reaction system as described in Additional file 5: Table S4. The OD value of each sample was measured by spectrophotometer (λ = 570 nm).

### Statistical analysis

Data were analyzed using GraphPad Prism 5 software [23]. Quantitative variables were expressed as means ± SEM and analyzed by one-way ANOVA, Student’s *t* test, Kruskal-Wallis test, or Mann-Whitney test. Qualitative variables were compared using Pearson’s χ^2^ test or Fisher exact test. The log-rank test was used to determine the statistical significance of the differences between metastasis-free survival curves. R/Bioconductor software was used for all bioinformatics analysis. Results were considered statistically significant at *p* < 0.05.

### Other materials and methods

Details on Western blotting, RNA extraction, real-time PCR [24, 26], RNA immunoprecipitation and sequencing [27], luciferase reporter assay [28], miRNA in situ hybridization, patient selection and tissue microarray (TMA) construction [29], immunofluorescence staining, immunohistochemical staining [30], and functional assays, such as migration, and Matrigel invasion assays [19], wound healing assays [31], in vivo assays for tumor metastasis [32, 33], cell membrane fluidity evaluated by TMA-DPH, statistical analysis [28], are described in the Additional file 6.

## Results

### Low endogenous miR-612 level correlates with poor outcome of patients

In our previous study, we have noticed that the level of miR-612 had a significant inversed correlation with tumor size and stage, intrahepatic metastasis, and microvascular invasion in one cohort with 37 primary HCC tissues. And the patients with low expressed levels of miR-612 usually had a poor metastasis-free survival [19]. To confirm above observation, we enrolled another cohort with 134 primary HCC tissues, whose clinicopathological features are listed in details in Table 1.

**Table 1:** Correlation between miR-612 expression and clinicopathologic in HCC patients

Characteristics	Cases (*n* = 134)	Relative miR-612 expression		
		miR-612^low^ (*n* = 63)	miR-612^high^ (*n* = 71)	*p*-value^a^
Age (year)				0.284
≤ 58	70 (52.2%)	36	34	
> 58	64 (47.8%)	27	37	
Sex				0.790
Male	105 (78.4%)	50	55	
Female	29 (21.6%)	13	16	
AFP (ng/ml)				0.136
≤ 20	58 (43.3%)	23	35	
> 20	76 (56.7%)	40	36	
ALT(U/L)				0.789
≤ 75	115 (85.8%)	55	60	
> 75	19 (14.2%)	8	10	
HBsAg				0.101
Negative	32 (23.9%)	11	21	
Positive	102 (76.1%)	52	50	
HCVAb				0.320
Negative	12 (9.0%)	4	8	
Positive	122 (91.0%)	59	63	
Liver cirrhosis				0.647
No	32 (23.9%)	13	17	
Yes	101 (75.4%)	50	54	
NA	1 (0.7%)			
Tumor size (cm)				**0**.**002**
< 3	21 (15.7%)	5	16	
3–5	40 (29.9%)	14	26	
> 5	73 (54.4%)	44	29	
NA	0			
Tumor number				0.959
Single	96 (71.6%)	45	51	
Multiple	38 (28.4%)	18	20	
BCLC				**0**.**045**
A	14 (10.4%)	3	11	
B	71 (53.0%)	31	40	
C	49 (36.6%)	29	20	
NA	0			
Differentiation				**0**.**032**
I	4 (3.0%)	1	3	
II	89 (66.4%)	40	49	
III	41 (30.6%)	22	19	
NA	0			
Tumor thrombus				**0**.**020**
No	82 (61.2%)	32	50	
Yes	52 (38.8%)	31	21	
NA	0			
Ascites				0.553
No	129 (96.3%)	60	69	
Yes	5 (3.7%)	3	2	
NA	0			

Bold data indicates statistical significance (*p* < 0.05)

^a^Qualitative variables were compared using χ^2^-test

**Fig. 1:**
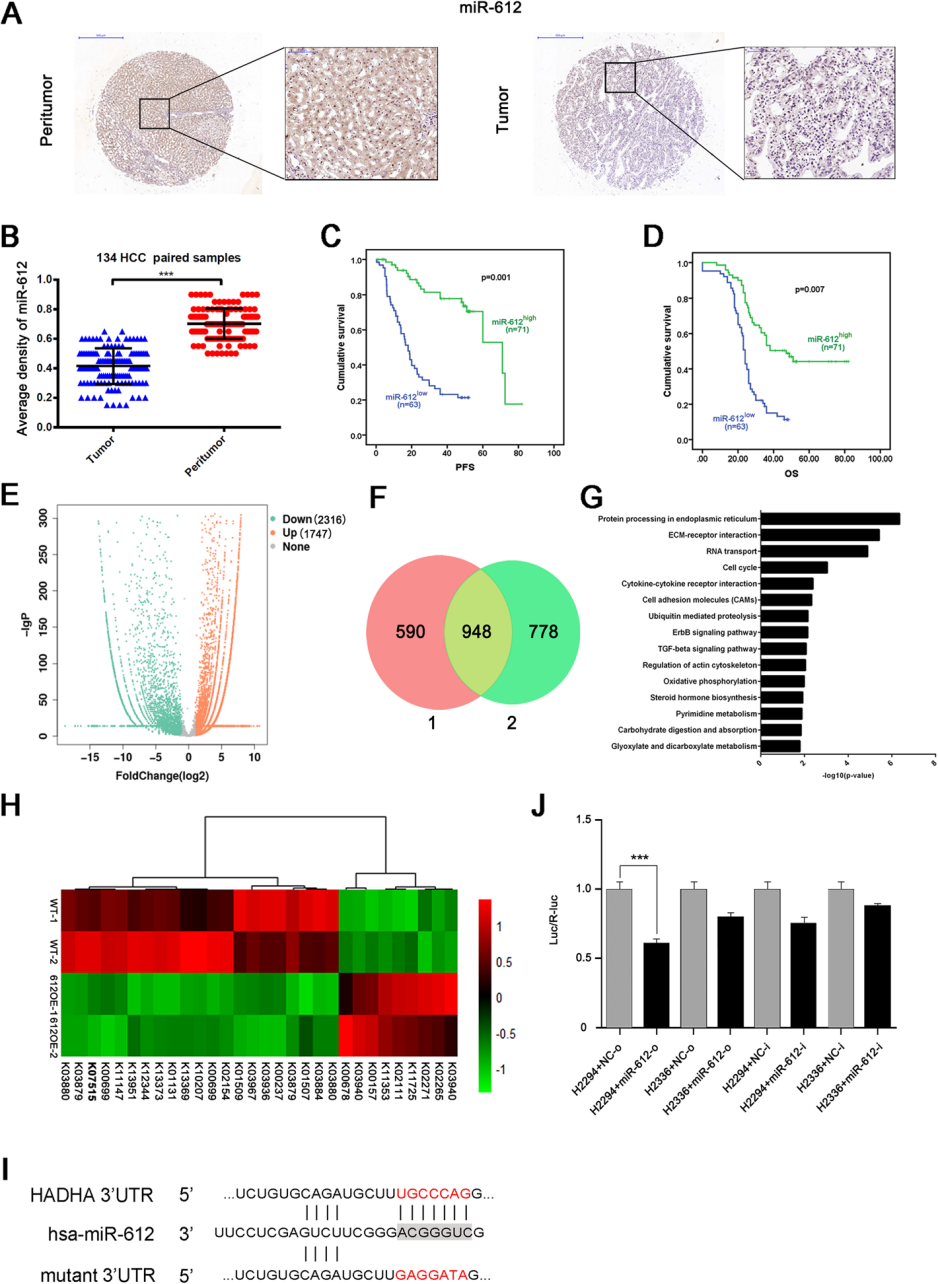
MiR-612 and *hadha*, its new target, in HCC. **a**, **b** Representative images with high and low expressed level of miR-612 in HCC and their paired adjacent tissues in immunohistochemistry. Bars: (left) magnification × 100, (right) magnification × 400. **c**, **d** Kaplan–Meier analysis of PFS and OS in HCC patients using SPSS 22.0. **e** Volcano map of the differential expressed genes (Ordinate: statistical significance of changes in expression; abscissa: multiple changes in the differential genes. Orange represents upregulated genes and downregulated ones in green). **f** Repeated downregulated genes in two independent RNA-seqs. **g** KEGG pathway analysis (Ordinate: the KEGG signal path; abscissa: *p* value). **h** Gene profiles related to lipid metabolism, including *hadha*. **i** Schematic diagram of the dual luciferase reporter vector of miRNA target. **j** Luciferase activity in 293T cells. H2294:pMIR-REPORT *hadha* WT 3′-UTR; H2336:pMIR-REPORT *hadha* mut 3′-UTR. Statistical analysis by Student’s *t* test. (***p < 0.001). Data are mean ± SEM of three independent experiments

Using fluorescence in situ hybridization, miR-612 was showed expressed both in cytoplasm and nuclei (Fig. 1a). The average density of miR-612 in cancerous tissues was significantly lower than these of paired adjacent non-HCC tissues (0.415 ± 0.010 vs. 0.702 ± 0.008, *p* < 0.001; Fig. 1b). When cutting off with median value of integrated optical density (mIOD), 63 of 134 patients (47%) were categorized into miR-612 low-expressed group (miR-612^low^) and the remaining (71/134, 53%) into miR-612 high-expressed group (miR-612^high^).

As shown in Table 1, miR-612 is negatively related with BCLC staging (*p* = 0.045), differentiation (*p* = 0.032), tumor size (*p* = 0.002), and tumor thrombus (*p* = 0.02) of HCC. Kaplan-Meier analysis showed that the low expressed levels of miR-612 were correlated with poor progress-free survival (PFS) (*p* = 0.001) and poor overall survival (OS) (*p* = 0.007; Fig. 1c, d), which was consistent with our previous results. Taken together, these clinical data reveal once more that low level of miR-612 is a poor prognostic predictor of HCC patients after surgical resection.

### *HADHA* is a direct target of miR-612

Loss- and gain-of-function studies showed that *akt2* and *sp1* are the two direct targets of miR-612 which negatively regulated cancerous metastasis and the stemness of HCC [19, 24]. As a given miRNA usually regulate multiple targets and signaling pathways simultaneously, miR-612-hybridized RNAs were pulled down and sequenced by RNA immunoprecipitation and RNA-seq assays respectively for a comprehensive targets picture of miR-612. After kicked off unknown genes, 1747 up-expressed genes and 2316 down-expressed genes were filtrated in miR-612-mimic transfected cells when compared with their negative counterpart (Fig. 1e). The gene ontology of these differentially miR-612-binding targets in biological process, cellular component, and molecular function was showed in Additional file 1: Figure S1D-F. In all down-expressed genes, 948 were identified twice by RNA-seq analyses (Fig. 1f), and their biological functions focus on the processes of endoplasmic reticulum, ECM-receptor interaction, RNA transport, actin cytoskeleton remodeling, as well as metabolism reprogramming according to Kyoto encyclopedia of genes and genomes (KEGG) analysis (Fig. 1g). More interestingly, *hadha* (KEGG: K07515), one pivotal center of miR-612 predicted network in our previous study, was repeatedly pulled-down, indicating the involvement of miR-612 in lipid metabolism (Fig. 1h). To test whether *hadha* is a direct target of miR-612, a wild-type (plasmids named H2294) and mutant (plasmids named H2336) of *hadha* at seed sequence in a 3′-UTR element were cloned into a dual luciferase reporter (Fig. 1i), and then transiently transfected into 293 T cells. Indeed, the levels of luciferase activities have been reduced nearly by 50% in cells transfected with wild-type 3′-UTR element of *hadha* when compared with cells with control vector (*p* < 0.001; Fig. 1j). In conclusion, *hadha* is the direct target gene of miR-612.

### MiR-612 suppresses HCC invasion and migration by targeting *HADHA*

**Fig. 2:**
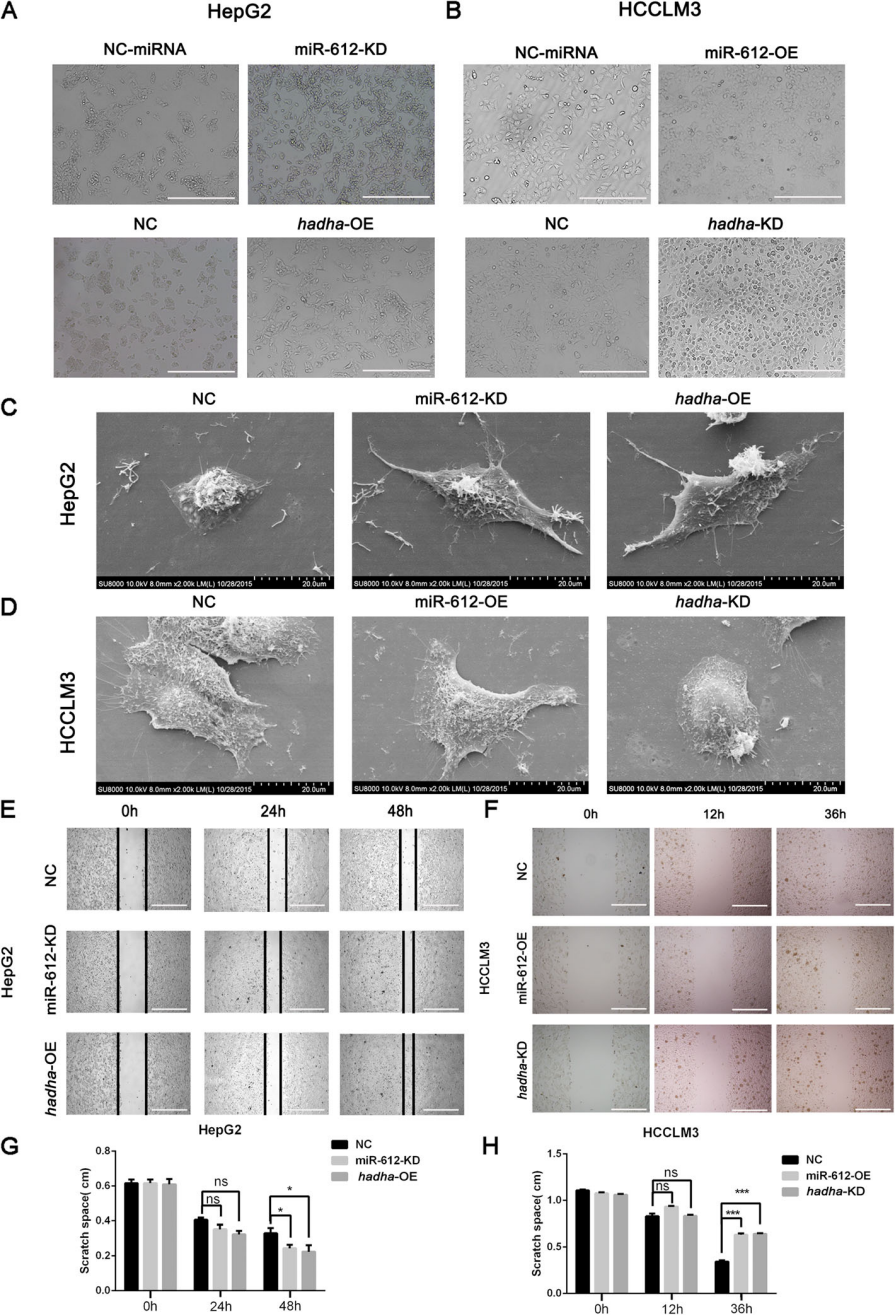
Morphology and mobility of HCC regulated by miR-612 and *hadha*. **a**, **b** Morphological changes of HepG2 and HCCLM3 infected with indicated lentivirus in microscope. Scale bars, 200 μm. **c**, **d** Morphological changes of HepG2 and HCCLM3 infected with indicated lentivirus in electron microscopy. **e**, **g** Wound healing of HepG2 infected with miR-612-KD or *hadha*-OE lentivirus. Scale bars, 200 μm. **f**, **h** Wound healing of HCCLM3 infected with miR-612-OE and *hadha*-KD lentivirus. Scale bars, 200 μm. (ns: not significant; *p < 0.05; ***p < 0.001). Data are mean ± SEM of three independent experiments

Although the inhibitory effects of miR-612 on HCC invasion and metastasis had been established [19], its underlying mechanisms was far from elucidated. In the study, we focus our attention on *hadha* in mediating the biological functions of miR-612. Therefore, loss- and gain-of-functions of miR-612 or *hadha* were investigated using a lentivirus infected strategy (Additional file 2: Figure S2A-C, miR-612-overexpression and -knockdown virus or respective mock were suppled by Hanyin Biotechnology Limited Company, Shanghai; the series virus of HADHA or negative control were ordered in Genechem Co., LTD, Shanghai). Remarkably morphological changes of HCCLM3 and HepG2 were noted after gain- or loss-of-function of miR-612 or *hadha*. First of all, HepG2^miR-612-KD^ or HepG2^*hadha*-OE^ cells appeared to grow in scattering spindle morphology rather than in ovoid cluster in NC cells in optical microscope (Fig. 2a). While the opposite morphological changes were observed in HCCLM3^miR-612-OE^ or HCCLM3^*hadha*-KD^ cells which tended to grew into pebble-shape (Fig. 2b). Indeed, more fusiform shapes with slender pseudopodia could be viewed in HepG2^miR-612-KD^ or HepG2^*hadha*-OE^ cells using scanning electron microscope than these of HepG2^NC^ cells (Fig. 2c), while more pseudopodia could be found at the edge of HCCLM3^NC^ cells than these of HCCLM3^miR-612-OE^ or HCCLM3^*hadha*-KD^ cells (Fig. 2d). These results showed clearly that miR-612 and *hadha* regulated HCC cell morphology oppositely.

**Fig. 3:**
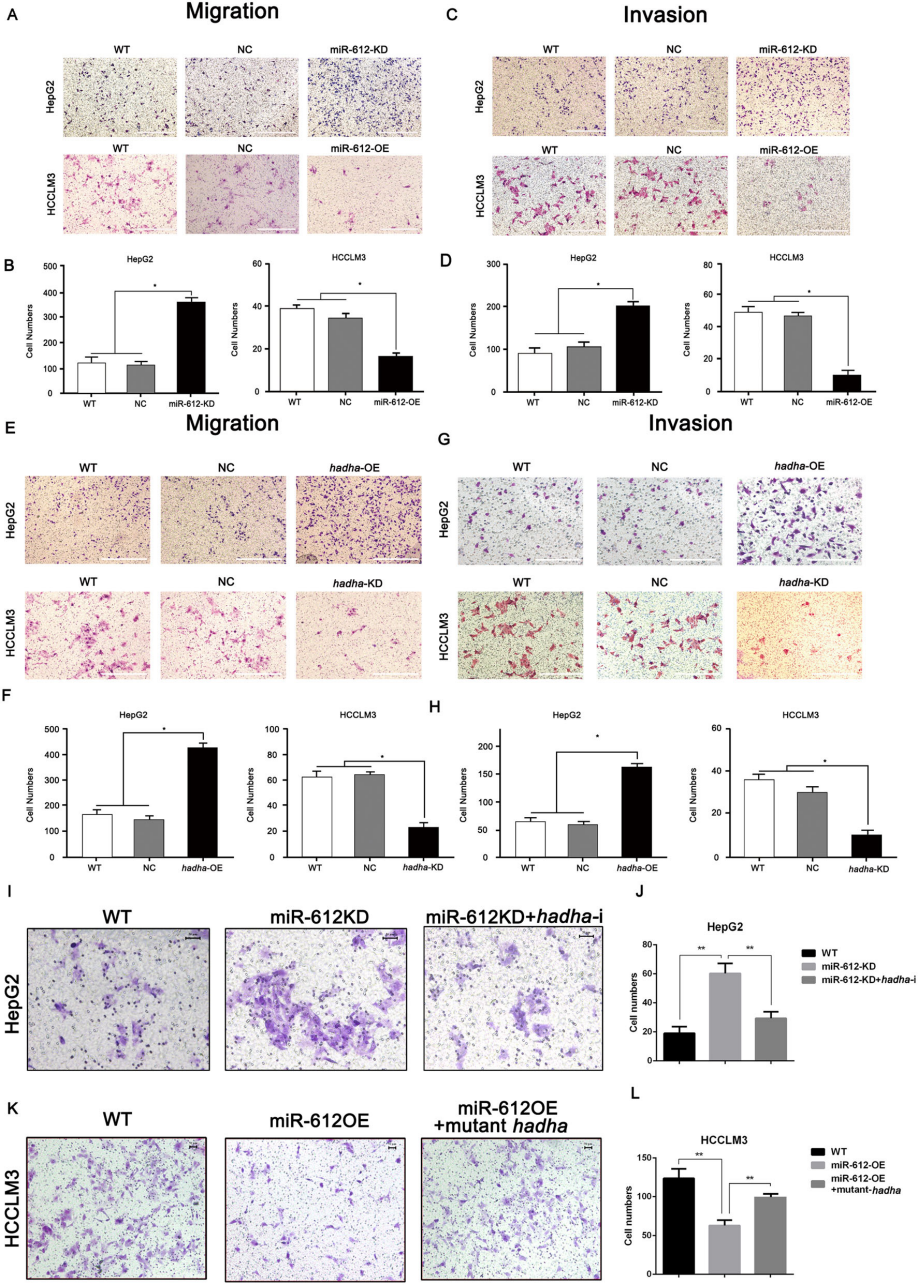
MiR-612 suppresses invasion and migration of HCC by targeting *hadha*. **a**, **b** Cell migration abilities and statistic results of HepG2^miR-612-KD^ and HCCLM3^miR-612-OE^ cells. Scale bars, 200 μm. **c**, **d** Cell invasion abilities and statistic results of HepG2^miR-612-KD^ and HCCLM3^miR-612-OE^ cells. Scale bars, 200 μm. **e**, **f** Cell migration abilities and statistic results of HepG2^*hadha*-OE^ and HCCLM3^*hadha*-KD^ cells. Scale bars, 200 μm. **g**, **h** Cell invasion abilities and statistic results of HepG2^*hadha*-OE^ and HCCLM3^*hadha*-KD^ cells. Cell migration abilities and statistic results of HepG2^miR-612-KD^ cells after *hadha* was knocked down. Scale bars, 200 μm. **i**, **j** Cell migration abilities and statistic results of HCCLM3 ^miR-612-OE^ cells after HADHA was rescued. Scale bars, 50 μm. **k**, **l** Cells (*p < 0.05; **p < 0.01). Data are mean ± SEM of three independent experiments. Scale bars, 50 μm

Next, we wondered whether *hadha* would promote cell migration and invasion of HCC, the key steps in tumor metastatic cascade, opposite to miR-612. Again, the scratched spaces reduced no difference at 24 h, but up to 30.5% at 48 h in HepG2^miR-612-KD^ (*p* = 0.018; Fig. 2e, g) and no difference at 24 h, up to 50% at 48 h in HepG2^*hadha*-OE^ respectively (*p* = 0.021; Fig. 2e, g). While the wounded spaces were not significant at 12 h, and increased nearly by 50% at 36 h both in HCCLM3^miR-612-OE^ (*p* < 0.001; Fig. 2f, h) or HCCLM3^*hadha*-KD^ cells (*p* < 0.001; Fig. 2f, h), compared with their control counterparts. Similarly, the numbers of HepG2^miR-612-KD^ or HCCLM3^miR-612-OE^ cells in Transwell assays were about 3- or 0.5-fold as much as their control cells respectively (*p* < 0.05; Fig. 3a, b). The numbers of HepG2^*hadha*-OE^ or HCCLM3^*hadha*-KD^ cells in Transwell assays were increased or decreased by two times compared with their corresponding NC cells (*p* < 0.05; Fig. 3e, f). Again, the numbers of successfully invaded HepG2^miR-612-KD^ or HepG2^*hadha*-OE^ cells were about 2- or 3-fold as much as nontreated (WT) and blank vector (NC) infected cells (*p* < 0.05; Fig. 3c–f). And the numbers of invaded HCCLM3^miR-612-OE^ or HCCLM3^*hadha*-KD^ cells were significantly reduced by more than 5- or 3-fold, respectively. (*p* < 0.05; Fig. 3c–f). The above results suggested that miR-612 inhibit cell migration and invasion of HCC probably by targeting *hadha*.

To further confirm the effects of HADHA in miR-612-mediated cell mobility, HADHA levels were largely rescued in HCCLM3^miR-612-OE^ cells after co-transfected with synonymous mutant of *hadha*, or inhibited by *hadha*-shRNA in HepG2^miR-612-KD^ cells. Indeed, 2-fold enhanced invasive abilities of HepG2^miR-612-KD^ cells were partially compromised after co-transfected with *hadha*-shRNA (*p* < 0.01; Fig. 3i, j). Similarly, decreased invasive abilities of HCCLM3^miR-612-OE^ cells were partially restored when co-transfected with synonymous mutant of *hadha* (*p* < 0.01; Fig. 3k, l). All these findings demonstrated that miR-612 did inhibit cell migration and invasion partially mediated by HADHA.

### MiR-612 restrains invadopodia of HCC directly by HADHA and inhibit EMT via Wnt/β-catenin signaling

**Fig. 4:**
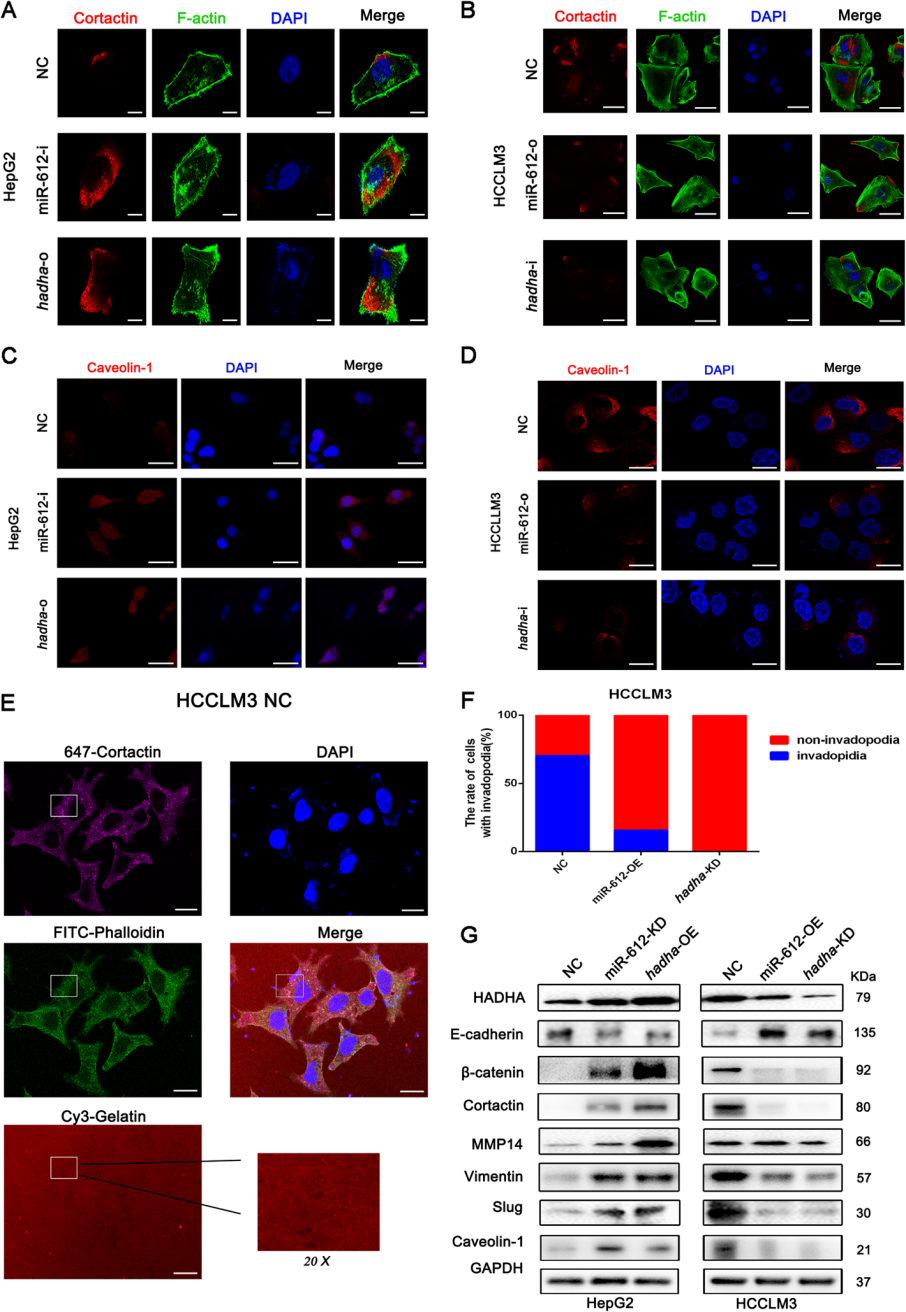
MiR-612 restrains HCC invadopodia by HADHA. **a**, **c** Expression of invasive pseudopodia-related proteins, Cortactin, F-actin, and Caveolin-1, in HepG2 cells with indicated treatments. **a** Scale bars, 5 μm. **c** Scale bars, 30 μm (**b**, **d**) Expression of invasive pseudopodia-related proteins, Cortactin, F-actin and Caveolin-1, in HCCLM3 cells with indicated treatments. **b** Scale bars, 50 μm. **d** Scale bars, 15 μm. **e**, **f** The number and statistic results of invasive pseudopods of HCCLM3 cells. Scale bars, 15 μm. **g** EMT and invadopodia biomarkers in HepG2 and HCCLM3 cells with indicated treatments analyzed by Western blots

Our morphological observations of electron microscopy suggested that invadopodia may be involved in miR-612 regulation on HCC metastatic potential. Naturally, Cortactin and Caveolin-1, two vital proteins of invadopodia, were selected to analyze their conversion after miR-612 or *hadha* manipulation by laser confocal microscopy [34, 35]. Cortactin (red staining), F-actin (green staining) (Fig. 4a, b), and Caveolin-1 (red staining) (Fig. 4c, d) were expressed much sharper in HCCLM3^NC^ cell with lower endogenous levels of miR-612 than these in HepG2^NC^ cell. However, Cortactin, F-actin, and Caveolin-1 were enhanced robustly both in HepG2^miR-612-i^ cells and HepG2^*hadha*-o^ cells compared to HepG2^NC^cells, and these proteins, especially Cortactin, were gathered at the edge of the cells (Fig. 4a, c). While, all these proteins were significantly weakened in HCCLM3^miR-612-o^ and HCCLM3^*hadha*-i^ cells (Fig. 4b, d), which were correlated to their decreased abilities of cell migration and invasiveness previously. These results showed intuitively that miR-612 and HADHA could remodel F-actin cytoskeleton and invadopodia formation of HCC via dynamic conversations of Cortactin and Caveolin-1.

Invadopodia is usually resorted to degrade the surrounding matrix and promote a distant invasion of cancerous cell. Therefore, the impacts of miR-612 and *hadha* on functional invadopodia were further tested by gelatin degradation assay using HCCLM3, HCC cell line with high metastatic potential. The function of invadopodia was judged by the co-localizations of matrix degradation, F-actin puncta, and Cortactin foci according to the manufacturer’s instructions, as shown in the white rectangles in HCCLM3^NC^ cells (Fig. 4e, f). Only a small amount of Cortactin and F-actin could be observed without obvious gelatin degradation, indicating that they were in a non-functional status of invadopodia in HCCLM3^miR-612-OE^ and HCCLM3^*hadha*-KD^ cells (Additional file 2: Figure S2D, E). The above results clearly showed that the inhibitory effects of miR-612 and the promoting effects of *hadha* were exhibited corresponding to the numbers of functional invadopodia.

To further clarify regulatory proteins of invadopodia after miR-612 or *hadha* manipulation, a series of proteins whose expressed levels were detected by Western blottings. And Cortactin and Caveolin-1 were once again low expressed in HepG2^NC^ cells and high expressed in HCCLM3^NC^ cells. Both proteins were dramatically increased in HepG2^miR-612-KD^ or HepG2^*hadha*-OE^ cells and markedly decreased in HCCLM3^miR-612-OE^ or HCCLM3^*hadha*-KD^ cells, which was consistent with the numbers of functional invadopodia and the migration abilities of cancerous cells.

In order to explore whether miR612 /HADHA would suppress the invadopodia formation and subsequently rearrange the MMPs and reduce other adhesion molecules accumulation thereby inhibit epithelial to mesothelial transformation (EMT), we detected miR-612 and hadha manipulation expression change on EMT-associated signal molecules. E-cadherin, an epithelial phenotype protein, and β-catenin, a mesothelial phenotype protein, were significantly down- and upregulated respectively in HepG2^miR-612-KD^ or HepG2^*hadha*-OE^ cells. Meantime, knockdown of miR-612 or overexpression of hadha in HepG2 induced an augment in the protein expression of Vimentin, Slug and MMP14. The opposite trends were observed in HCCLM3^miR-612-OE^ or HCCLM3^*hadha*-KD^ cells. (Fig. 4g). These results indicated that HADHA promoted the formation of functional invadopodia against miR-612, accompanied by the EMT of HCC through Wnt/β-catenin signaling pathway [19]. The formation of invadopodia and occurrence of EMT jointly promote HCC cell metastasis and invasion.

### HADHA promotes HCC metastasis in vivo

**Fig. 5:**
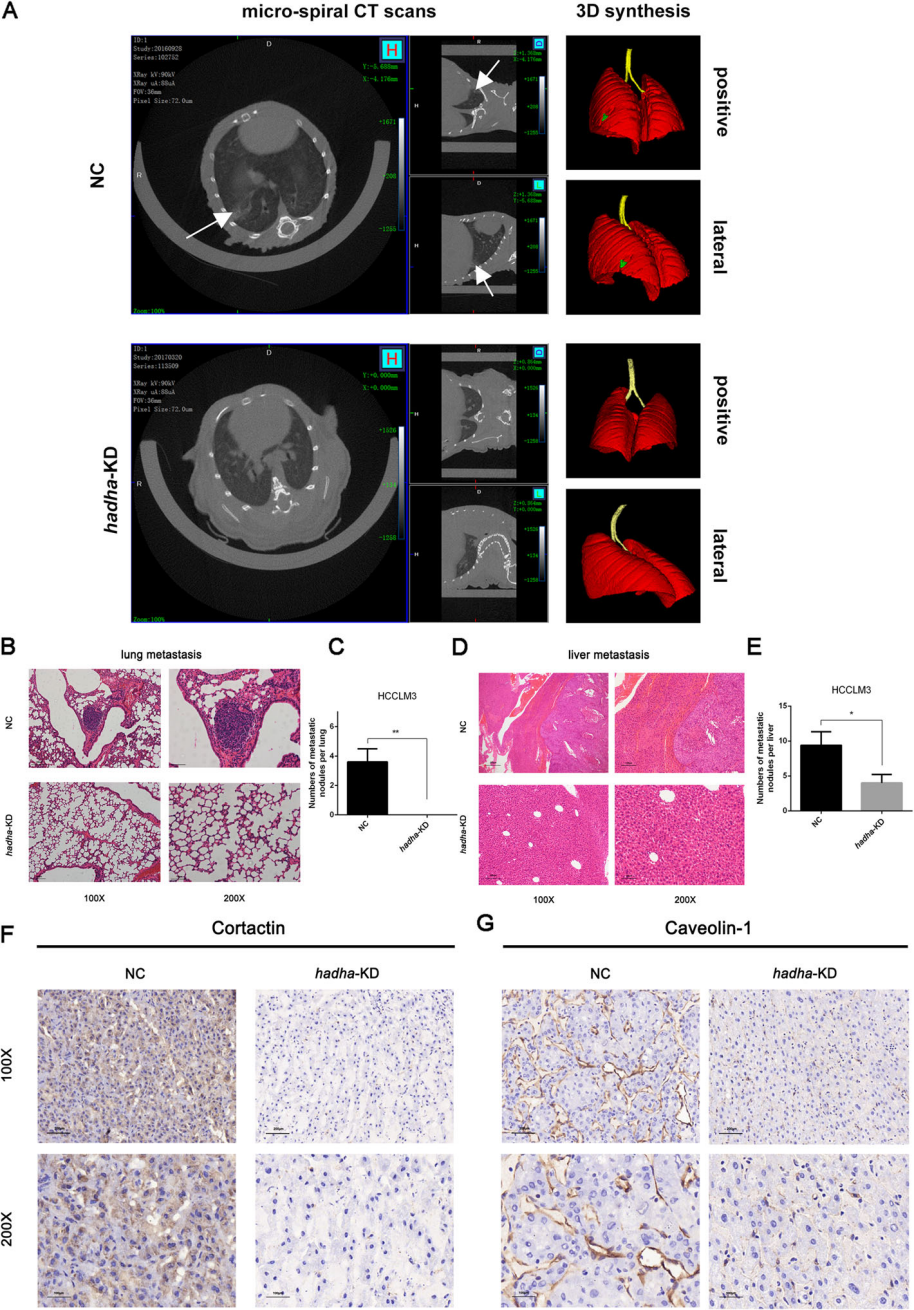
HADHA promotes metastasis of HCC in vivo. **a** Metastatic foci in lung were imaged by micro-spiral CT scans using 3D synthesis. **b**, **c** Metastatic foci in lung and their statistic results of indicated HCCLM3 orthotopic xenografts (HCCLM3^*hadha*-KD^ or HCCLM3^NC^) in H-E staining. **d**, **e** Metastatic foci in liver and their statistic results of indicated HCCLM3 orthotopic xenografts in H-E staining. Bars: (left) magnification × 100, scale bars, 200 μm. (right) magnification × 200, scale bars, 100 μm. **f**, **g** The levels of Cortactin and Caveolin-1 in liver orthotopic xenografts using IHC analyses. Bars: (up) magnification× 100, scale bars, 200 μm. (down) magnification × 200. scale bars, 100 μm. (*p < 0.05; **p < 0.01)

In our previous study, miR-612 had been verified to suppress the invasive-metastatic cascade in HCC in vitro and in vivo [19]. Here, the roles of HADHA on in-vivo tumor growth and metastasis were surveyed using micro-spiral CT scanning in the orthotopic HCCLM3 xenograft models (Additional file 3: Figure S3A, B). After 8 weeks, we observed that two mice in HCCLM3^NC^ group (2/6, one died of ascites) developed pulmonary metastasis foci compared with none of HCCLM3^*hadha*-KD^ group (0/6) (Fig. 5a). After sacrificed, obviously smaller tumor volumes and less intraliver metastasis foci were found in HCCLM3^*hadha*-KD^ xenografts than in HCCLM3^NC^ xenografts (Additional file 3: Figure S3C). The numbers of metastatic foci in lung and liver of HCCLM3^*hadha*-KD^ recipient mice were dramatically lower than that of HCCLM3^NC^ in HE staining (*p* < 0.01, *p* < 0.05; Fig. 5b–e). Meanwhile, Cortactin, mainly located in cytoplasm, was significantly downregulated in paraffin-embedded HCCLM3^*hadha*-KD^ xenograft tissues. And Caveolin-1, mainly expressed on membrane, was also remarkably decreased in paraffin-embedded HCCLM3^*hadha*-KD^ xenograft tissues using immunological histological chemistry (IHC) staining (Fig. 5f, g). These observations demonstrated that HADHA itself indeed promoted the formation of invadopodia in vivo, resulting in an accelerated distant metastasis in HCC xenograft models.

### HADHA promotes β-oxidation of fatty acids in HCC

**Fig. 6:**
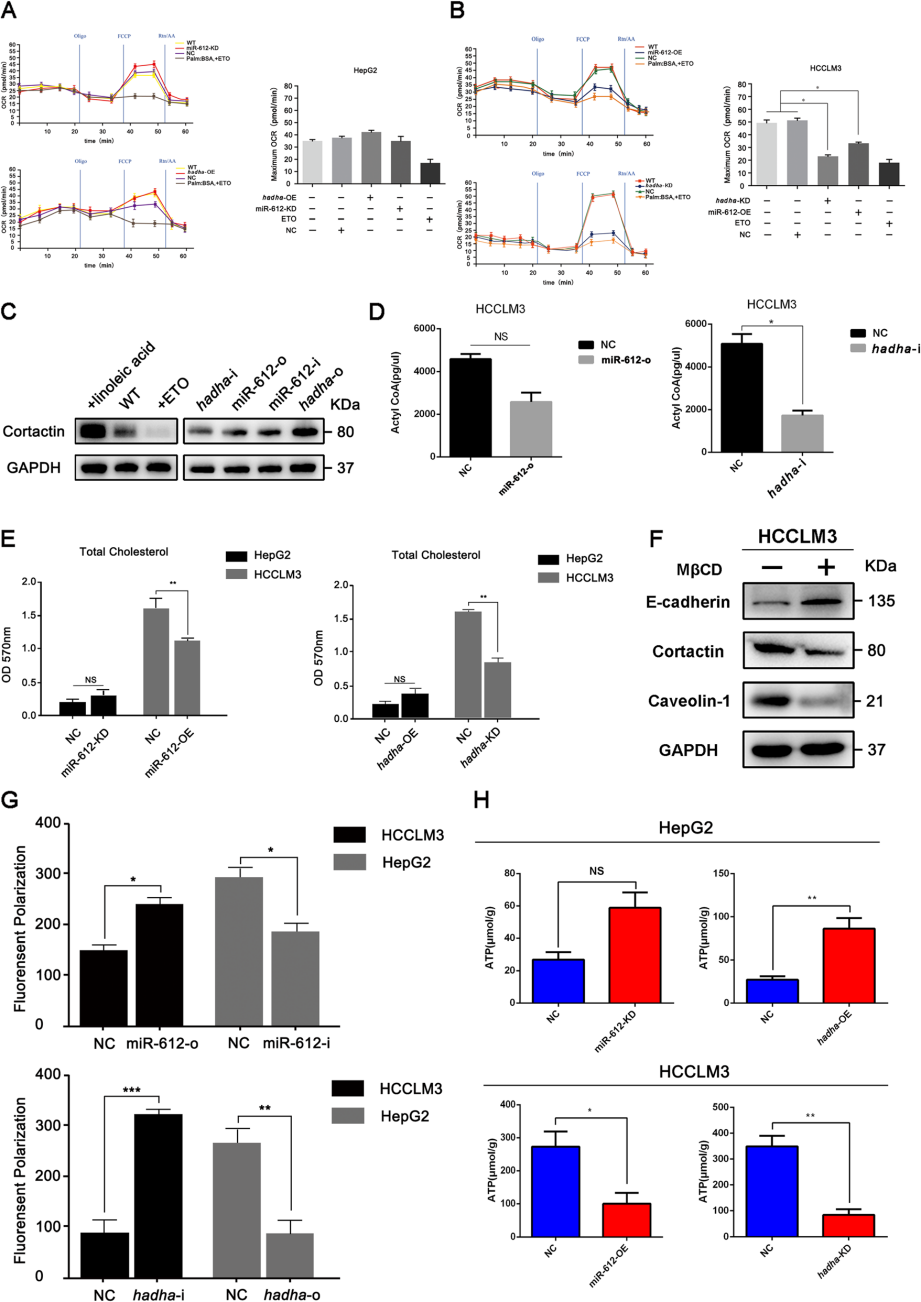
HADHA promotes β-oxidation of fatty acids in HCC. **a** OCR, the activities of β-oxidation, in HepG2 cells treated with 100 μM ETO, as well as in HepG2^miR-612-KD^, HepG2^*hadha*-OE^, and HepG2^NC^ cells. **b** OCR, the activities of β-oxidation, in HCCLM3 cells treated with 100 μM ETO, as well as in HCCLM3^miR-612-OE^, HCCLM3^*hadha*-KD^, and HCCLM3^NC^ cells. **c** The protein levels of Cortactin in HCCLM3 cells treated with 100 μM ETO or 100 mM linoleic acid, as well as in HCCLM3^miR-612-o^, HCCLM3^miR-612-i^, HCCLM3^*hadha*-o^, and HCCLM3^*hadha*-i^ cells. **d** The intracellular levels of acetyl CoA in HCCLM3^miR-612-o^, HCCLM3^*hadha*-i^, and HCCLM3^NC^ cells. **e** The cellular levels of cholesterol in HepG2^miR-612-KD^, HCCLM3^miR-612-OE^, HepG2^*hadha*-OE^, and HCCLM3^*hadha*-KD^ cells. **f** The protein levels of Cortactin, Caveolin-1, E-cadherin, and GAPDH in HCCLM3 cells treated with or without 1 mM MβCD. **g** Fluorescence intensity in HepG2^miR-612-i^, HCCLM3^miR-612-o^, HepG2^*hadha*-o^, and HCCLM3^*hadha*-i^ cells detected by fluorescence spectrophotometer (Ex/Em = 360/460 nm). Fluorescence polarization is calculated by the formula (**h**) The levels of ATP in HepG2^miR-612-KD^, HCCLM3^miR-612-OE^, HepG2^*hadha*-OE^, and HCCLM3^*hadha*-KD^ cells analyzed by spectrophotometer (λ = 636 nm). (NS: no significance; *p < 0.05; **p < 0.01; ***p < 0.001). Data are mean ± SEM of three independent experiments

To meet aggressive tumor growth, cancerous cells usually resort to seeking other resources, such as fatty acids, for material and energy supply apart from glycolysis even in normoxia. HADHA catalyzes the last three steps of mitochondrial β-oxidation of long chain fatty acids [36]. Here, we wondered whether or not HADHA regulated HCC invasion and metastasis by lipid reprogramming. Using bioenergetic energy analyzer, the activity of β-oxidation of fatty acids can be perfectly gauged by oxygen consumption rate (OCR). As etomoxir (ETO) is a specific inhibitor of β-oxidation by blocking the activity of carnitine palmitoyl transferase (CPT-1), it was chosen as a positive control in the study [37]. Indeed, the OCR of 100 μM ETO-treated HepG2 and HCCLM3 cells were both decreased nearly by 50% when compared to their corresponding untreated cells (Fig. 6a, b). The maximum OCR of HepG2^miR-612-KD^ and HepG2^*hadha*-OE^ cells increased slightly, but no significant difference from HepG2^WT^ and HepG2^NC^ cells (*p* > 0.05; Fig. 6a). In contrast, the OCR of HCCLM3^miR-612-OE^ and HCCLM3^*hadha*-KD^ cells were decreased obviously. Compared with the levels of HCCLM3^WT^ and HCCLM3^NC^ cells, the maximum OCR has reduced by 40% in HCCLM3^miR-612-OE^ and by 60% in HCCLM3^*hadha*-KD^ (*p* < 0.05; Fig. 6b). All these results suggested that miR-612 could inhibit β-oxidation of fatty acids by downregulating the expression of HADHA in HCC, especially in HCCLM3 cell.

To verify the role of HADHA-mediated β-oxidation on invadopodia and HCC metastasis, the levels of Cortactin were blotted after treated with 100 μM ETO [37] and 100 mM linoleic acid, a β-oxidation agonist. Unsurprisingly, the level of Cortactin was significantly upregulated in linoleic acid-treated HCCLM3 cells and downregulated in ETO-treated cells (Fig. 6c). And Cortactin was reduced remarkably in HCCLM3^*hadha*-i^. Correspondingly, the levels of Cortactin were significantly increased both in HCCLM3^miR-612-i^ and HCCLM3^*hadha*-o^. These results indicated that the increased activity of HADHA-mediated β-oxidation did promote the formation of invadopodia in HCC cells.

To evaluate the effects of miR-612 and HADAH on acetyl-CoA and ATP syntheses, acetyl-coenzyme assay kit was applied according to the manufacture’s protocol. The standard line of acetyl CoA was established before sample fluorometric detection in Additional file 3: Figure S3D. The acetyl-CoA level of HCCLM3^miR-612-o^ cells was decreased by 1/3 of HCCLM3^NC^ cells, but no statistical significance between them (*p* > 0.05). More than 50% reduction of acetyl-CoA level was observed in HCCLM3^*hadha*-i^ cells (*p* < 0.05; Fig. 6d). Although opposite effects were achieved, no significant increased level of acetyl-CoA was observed both in HepG2^miR-612-i^ or HepG2^*hadha*-o^ cells (data not shown). Meanwhile, the level of ATP had been increased by 53% in HepG2^miR-612-KD^ (*p* > 0.05) and by 71% in HepG2^*hadha*-OE^ cells (*p* < 0.01; Fig. 6h), but reduced to 1/2 and 1/4 in HCCLM3^miR-612-OE^ (*p* < 0.05) and HCCLM3^*hadha*-KD^ cells respectively (*p* < 0.01; Fig. 6h). These results revealed that less level of acetyl-CoA and ATP was available for HCC growth and metastasis when β-oxidation inhibited by miR-612. However, the process could be largely reversed after the rescued expression of HADHA.

### HADHA-mediated cholesterol alteration directly affect cell membrane fluidity of invadopodia

It is well known that acetyl-CoA and ATP can be used for biosynthesis of cholesterol, which is an essential component to maintain membrane integrity and fluidity of animal cells, especially of invaginated caveolae [38, 39]. Membrane lipid rafts construction, especially reprograming of cholesterol metabolism, will influence the invadopodia formation and its function, subsequently affecting tumor cell metastasis. To investigate the role of HADHA on cholesterol biosynthesis in HCC cells, 1 mM methyl beta cyclodextrin (MβCD) was recruited to remove the cholesterol from plasma membrane [40]. With cholesterol assay kit, the endogenous cholesterol level of HepG2 was found to be much lower than that of HCCLM3 (Fig. 6e). And the levels of cholesterol were reduced by nearly 1/3 in HCCLM3^miR-612-OE^ (*p* < 0.01) and a half in HCCLM3^*hadha*-KD^ cells (*p* < 0.01) when compared with HCCLM3^NC^ cells (Fig. 6e).

Simultaneously, both levels of Cortactin and Caveolin-1 were decreased significantly accompanied with E-cadherin up-regulation (Fig. 6f) and cell morphology changed from a fusiform into a rounded shape in MβCD-treated HCCLM3 (data not shown). The fluorescence polarization (FP) was increased by 37.5% in HCCLM3^miR-612-o^ (*p* < 0.05), 68.4% in HCCLM3^*hadha*-I^ (*p* < 0.001) and decreased by more than 1/3 in HepG2^miR-612-i^ (*p* < 0.05), 2/3 in HepG2^*hadha*-o^ cells (*p* < 0.01; Fig. 6g), indicating that cell membrane fluidity was decreased by miR-612 and increased by HADHA. From the above results, we found that the levels of cholesterol oppositely regulated by miR-612 and HADHA, and decreased level of miR-612 could promote cholesterol biosynthesis and cell fluidity of HCC mediated by HADHA.

**Fig. 7:**
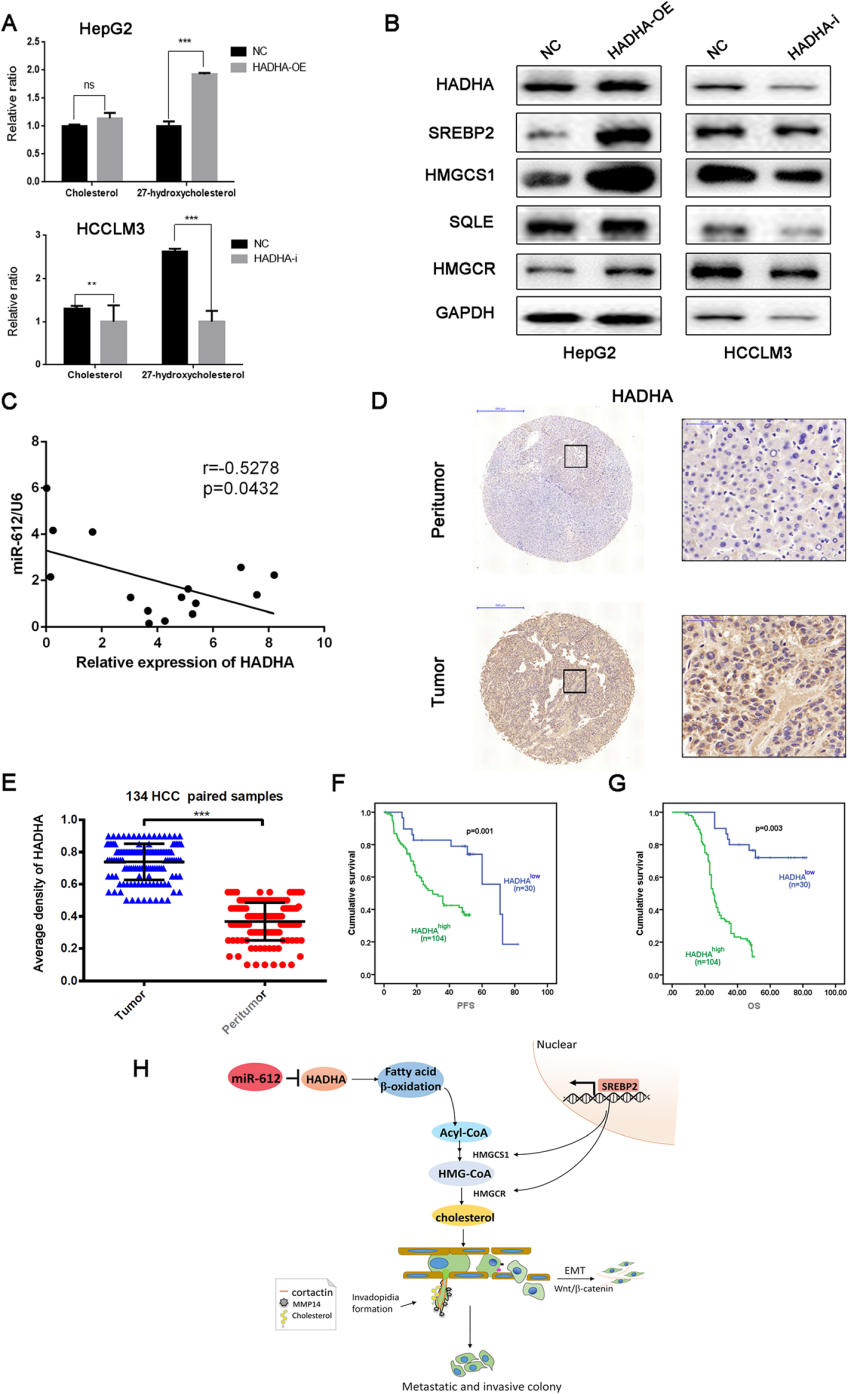
Clinical significance of HADHA in HCC patients. **a** The cholesterol and 27-hydroxycholesterol of HepG2 and HCCLM3 cells relative quantification based on liquid chromatography mass spectrometry (LC-MS)/MS system. (NS: no significance; **p < 0.01; ***p < 0.001). **b** Immunoblotting of proteins involved in cholesterol-biosynthesis pathway in HepG2^*hadha*-OE^, HCCLM3^*hadha*-KD^ cell, and their negative control cells. (Cells treated with negative control lentivirus). **c** Negative correlation between miR-612 and *hadha* mRNA in 15 HCC and their adjacent normal tissues. **d** Representative images of negative and positive HADHA staining in tumor and peritumor tissue from one HCC patient. Bars: (left) magnification × 100, scale bars, 500 μm (right) magnification × 400, scale bars, 50 μm. **e** The average densities of HADHA in 134 HCC and their paired normal tissues. **f**, **g** Kaplan-Meier analyses of PFS and OS in HCC patients using SPSS 22.0. **h** Hypothesis diagram of lipid reprogramming in HCC cells modulated by miR-612/HADHA/cholesterol axis

To further verify the HADHA-mediated HCC cell cholesterol content, we detected the lipid metabolites based on liquid chromatography mass spectrometry (LC-MS)/MS system. The results showed HepG2 ^hadha-OE^ had a high expression level of total cholesterol, especially 27-hydroxycholesterol, while HCCLM3 ^hadha-i^ presented a much lower expression (Fig. 7a).

SREBPs are transcription factors that are induced during cellular lipid deficit and that upregulate genes involved in cholesterol and fatty acid synthesis and trafficking. So we detected SREBP2 expression as well as its downstream targets to explore whether SREBP2/HMGCR cascade is the major regulator of cholesterol levels in HCC cells. The data showed that translocation of SREBP2 into the nucleus, and the ensuing transcription of sterol-responsive genes including HMGCR, 3-hydroxy-3-methylglutaryl-CoA synthase 1 (HMGCS1), mevalonate diphosphate decarboxylase (MVD), and SQLE, thereby facilitating the cholesterol synthesis and changing the invadopodia cell membrane fluidity (Fig. 7b).

### Clinical significance of HADHA in HCC patients

To investigate the relationship of miR-612 and HADHA in HCC patients, total RNAs were first extracted from 15 HCC patients randomly and then the levels of miR-612 and *hadha* was analyzed by RT-PCR. There existed a significant negative correlation between miR-612 and *hadha* in HCC tissues, which was in line with previous observations (*r* = − 0.5278, *p* = 0.0432; Fig. 7c). To investigate the clinical significance of HADHA in HCC patients, 134 tumor tissues and paired peritumor tissues were prepared for HADHA level analysis using IHC. HADHA, mainly located in cytoplasma, expressed prominently high in liver cancer tissues than in peritumors tissues (*p* < 0.001; Fig. 7d, e). When cutting off with mIOD, 22.4% (30/134) of HCC patients were sorted into HADHA low-expressed group (HADHA^low^) and 77.6% (104/134) were into HADHA high-expressed group (HADHA^high^). Kaplan-Meier analysis showed that the high expressed levels of HADHA were correlated with poor OS (*p* = 0.003), and poor PFS (*p* = 0.001; Fig. 7f,g). In short, HADHA was a poor prognostic indicator and a potential biomarker of relapse in HCC patients after surgical resection.

Above all, our study revealed that the miR-612 could suppress the invadopodia formation, EMT process, and metastasis through the HADHA/acetylCoA/HMGCoA/cholesterol axis in HCCs (Fig. 7h).

## Discussion

Invadopodia is a kind of pseudopodia, which is mainly responsible for extracellular matrix degradation, cell migration and invasion in local, extravasation of blood vessels, and dissemination into a distant organ in a spatial and temporal manner [8, 41]. Therefore, a variety of components, such as Cortactin, Caveolin-1, Tks4/5, and protein tyrosine kinase Src of invadopodia, are usually used as indicators to gauge the mobility and invasion potentials of cancer cells in vitro and in vivo system [42, 43]. For example, Cortactin, a monomeric cytoplasmic protein, is able to be polymerized and rearranged in actin cytoskeleton of cell cortex after activated by external stimuli, thus promoting the formation of invadopodia, cell migration, and metastasis [44]. Caveolin-1, a scaffolding protein, can interact with Src tyrosine kinases, cholesterol, and TGF-β receptor, etc., during the formation of invadopodia [34, 35]. By now, invadopodia is regarded as a kind of microdomain on cell membranes which is rich of cholesterol. If the cholesterol was depleted, invadopodia formation and persistence could be impaired. On these microdomains, a lot of proteins such as Cortactin, Caveolin-1, Receptors, signaling adaptors, and trafficking proteins are assembled. And thus, depletion of cholesterol will impair the membrane fluidity and protein trafficking of invadopodia [35]. Therefore, Cortactin, Caveolin-1, and cholesterol were chosen as the surrogated parameters for evaluating the formation and the function of invadopodia in the study. As to the subtle relationship among the cholesterol, invadopodia, and EMT, our results should be original. MiR-612/HADHA/cholesterol alteration will directly affect the lipid rafts construction, thereby influence the formation of invadopodia and invadopodia-mediated cellular matrix degradation, blunting EMT process of HCC. Furthermore, we discovered cholesterol metabolite, 27-hydroxycholesterol level change was shocking in HADHA-treated HCC cells. As reported, it could not only regulate the target genes controlling cholesterol, glucose, and fatty acid metabolism of liver but also increase the proliferation of various cancer cell [45, 46].

It is well known that biological processes of cells are largely dependent on energy, saying ATP, which is usually derived from the metabolisms of carbohydrates, lipids, and amino acids. In normal, ATP is mainly derived from acetyl-CoA of aerobic oxidation of glucoses through tricarboxylic acid cycle. However, in tumor cells, glucoses are often catalyzed into pyruvate through hexokinase (HK) and phosphofructokinase (PFK) pathways for de novo biosyntheses of lipids and amino acids to meet their uncontrolled growth and metastasis, and only a small portion are reduced into lactic acids for ATP synthesis in mitochondria [3]. As a result, lipid metabolism reprogramming, such as β-oxidation of fatty acids in mitochondria, is initiated rapidly as a compensatory pathway in these hypermetabolic cancerous cells to fuel cell proliferation, invasion, and metastasis [47] and protect tumor cells from anoikis as well [48]. Meanwhile, acetyl-CoA, a vital intermediate of β-oxidation of fatty acids, can either be used to synthesize ATP, or cholesterol, or so on, in mammal cells.

Although the dynamics of protein components, interactions, modifications, signaling, and functions during cytoskeletal remodeling in the processes of invadopodia have been extensively studied for more than 30 years [49], the roles of lipid metabolism reprogramming on invadopodia are still in early stage. In virtue of living cell imaging, invadopodia was recently confirmed as a special microdomain on membrane enriched with phospholipid PI(4,5)P2 and cholesterol. Our results also revealed that upregulation of cholesterol significantly increase membrane fluidity accompanied with Cortactin aggregation, suggesting that lipid metabolism is involved in the formation of invadopodia, and thus promote tumor cell metastasis [50, 51]. These results might open up a new perspective of lipid metabolism on tumor metastasis.

MiRNAs have been confirmed as important players in HCC metastasis using post-transcriptional mechanism [19]. In our previous studies, we revealed that miR-612 had pleiotropic inhibitory effects on cell proliferation, EMT, stemness, and metastasis of HCC via directly suppressing *akt2* expression [19]. In the present study, we found that *hadha* is another pivotal gene in miR-612 target network by RNA immunoprecipitation, RNA-seq, and luciferase report assay. A large amount of acetyl-CoA and ATP are produced by β-oxidation of long-chain fatty acids [52], resulting in many cancerous functions, such as autophagy [53], apoptosis [54], and so on. Here, we report a novel function of miR-612 on HCC invadopodia. Upregulation of miR-612 can significantly reduce cholesterol level and membrane fluidity of HCC, meanwhile, remarkably reduced the expressed level and function of Cortactin and Caveolin-1 in HCC invadopodia. Similar results were observed in HCC cells treated with β-oxidation inhibitor. Moreover, Wnt signaling pathway is able to lead to an increase in synaptic Cortactin and associated with cytoskeleton organization. Our finding also indicates that Cortactin is downstream of Wnt signaling, and associated to other cancer type s[47]. Our findings confirmed that miR-612 suppressed migration and invasion of HCC partially by HADHA-mediating lipid reprogramming and inhibited the formation of invadopodia and Wnt/β-catenin-regulated EMT progression.

## Conclusion

In summary, our study revealed that miR-612 negatively regulated invadopodia formation, matrix degradation, EMT, and metastasis of HCC by HADHA-mediated lipid reprogramming. The decreased miR-612 in HCC cells resulted in HADHA upregulation and initiated β-oxidation activities of fatty acids to supply sufficient acetyl-CoA, ATP for cholesterol biosynthesis via SREBP2/HMGCR cascade, which consequently affected HCC metastasis via lipid rafts and invadopodia. Here, we provide another new evidence of miR-612 as a tumor suppressor microRNA in HCC. The issue is not only conducive to a comprehensive understanding of the role of miR-612 but also lays a theoretical foundation for metastatic HCC diagnosis and prevention based on miR-612.

## Supplementary information

**Additional file 1:.** Figure S1. (A) Target genes and signaling pathways of miR-612 predicted by bioinformatics analyses. Red spot stands for HADHA. (B) Quality distribution of RNA pulled down by biotin-labeled miR-612 for RNA-seq analyses. (C) Base distribution of RNA pulled down by biotin-labeled miR-612 for RNA-seq analyses. (D-F) GO analyses of differential down-regulated genes based on their molecular function, cellular component and biological process.

**Additional file 2: **Figure S2. (A) HepG2 and HCCLM3 cells infected with *hadha* overexpression or knockdown lentivirus respectively. WT represents wild type cell line without any treatment. NC samples means the cell lines treated with negative control lentivirus. Scale bars, 200 μm. (B and C) The mRNA and protein levels of HADHA were tested by real-time PCRs and Western blots. (D and E) Fluorescence images of invadopodia staining by 647(Cortactin) and FITC (phalloidin) in HCCLM3^miR-612-OE^ and HCCLM3^*hadha*-KD^ cells. (***p < 0.001). Data are mean ± SEM of three independent experiments. Scale bars, 15 μm.

**Additional file 3: **Figure S3. (A and B) Tumor sizes and statistic results of subcutaneous HCCLM3^NC^ and HCCLM3^*hadha*-KD^ xenografts. (C) Tumor sizes of HCCLM3^NC^ and HCCLM3^*hadha*-KD^ xenografts in liver. (D) Standard plots of Acetyl CoA (*p < 0.05).

**Additional file 4.** Exel file. The original data of mir-612-targeted genes available via RNA immunoprecipitation which up- or down-regulated significantly.

**Additional file 5: Table S1.** Primers sequences of PCR. Table S2. Reaction Mixes for Acetyl-Coenzyme A Assay (unit: μl). Table S3. ATP detection operation rules (unit: μl). Table S4. Reaction mixes for cholesterol quantitation assay (unit: μl).

**Additional file 6.** Other materials and methods.

## Data Availability

Not applicable.

## Abbreviations

AFP: Alpha-fetoprotein
CPT-1: Carnitine palmitoyl transferase-1
EMT: Epithelial to mesothelial transformation
ETO: Etomoxir
FISH: Fluorescence in situ hybridization
FP: Fluorescence polarization
HADHA: Hydroxyacyl-CoAdehydrogenase/3-ketoacyl-CoAthiolase/enoyl-CoA hydratase, alpha subunit
HCC: Hepatocellular carcinoma
H-E staining: Hematoxylin-eosin staining
HK: Hexokinase
HMGCR: 3-Hydroxy-3-methylglutaryl-CoA reductase
HMGCS1: 3-Hydroxy-3-methylglutaryl-CoA synthase 1
IHC: Immunological histological chemistry
IOD: Integrated optical density
KEGG: Kyoto encyclopedia of genes and genomes
LCHAD: Long-chain 3-hydroxyacyl-CoA dehydrogenase
miRNA: Micro-RNA
MVD: Mevalonate diphosphate decarboxylase
MβCD: Methyl-β-cyclodextrin
NA: Not available
NS: No significant difference
OCR: Oxygen consumption rate
OS: Overall survival
PFK: Phosphofructokinase
PFS: Progress-free survival
SQLE: Squalene epoxidase
TMA: Tissue microarray
TNM: Tumor-node-metastasis
