# Correction to: MiR-612 regulates invadopodia of hepatocellular carcinoma by HADHA-mediated lipid reprogramming

**DOI:** 10.1186/s13045-020-00875-5

**Published:** 2020-05-04

**Authors:** Yang Liu, Li-Li Lu, Duo Wen, Dong-Li Liu, Li-Li Dong, Dong-Mei Gao, Xin-Yu Bian, Jian Zhou, Jia Fan, Wei-Zhong Wu

**Affiliations:** 1 Liver Cancer Institute, Zhongshan Hospital, Key Laboratory of Carcinogenesis and Cancer Invasion, Ministry of Education, Fudan University, 180 Fenglin Road, Shanghai, 200032 China; 2 Department of Oral Maxillofacial-Head and Neck Oncology, Shanghai Ninth People’s Hospital, School of Medicine, Shanghai Jiao Tong University, Shanghai, China; 3 Department of Head and Neck Surgery, Fudan University Shanghai Cancer Center, Shanghai, 200032 China; 4 Department of Radiation Oncology, Shanghai General Hospital, Shanghai Jiaotong University, Shanghai, 200080 China; 5 Department of Liver Surgery and Transplantation, Zhongshan Hospital, Fudan University, Shanghai, 200032 China

**Correction to: Journal of Hematology & Oncology (2020) 13:12**

**https://doi.org/10.1186/s13045-019-0841-3**

The original article [1] contains an error in Fig. 3c and Fig. 3g whereby the image of group WT and NC in HCCLM3 cell lines were repeated. The correct presentations of Fig. 3c and Fig. 3g can be seen ahead.

**Fig. 3:**
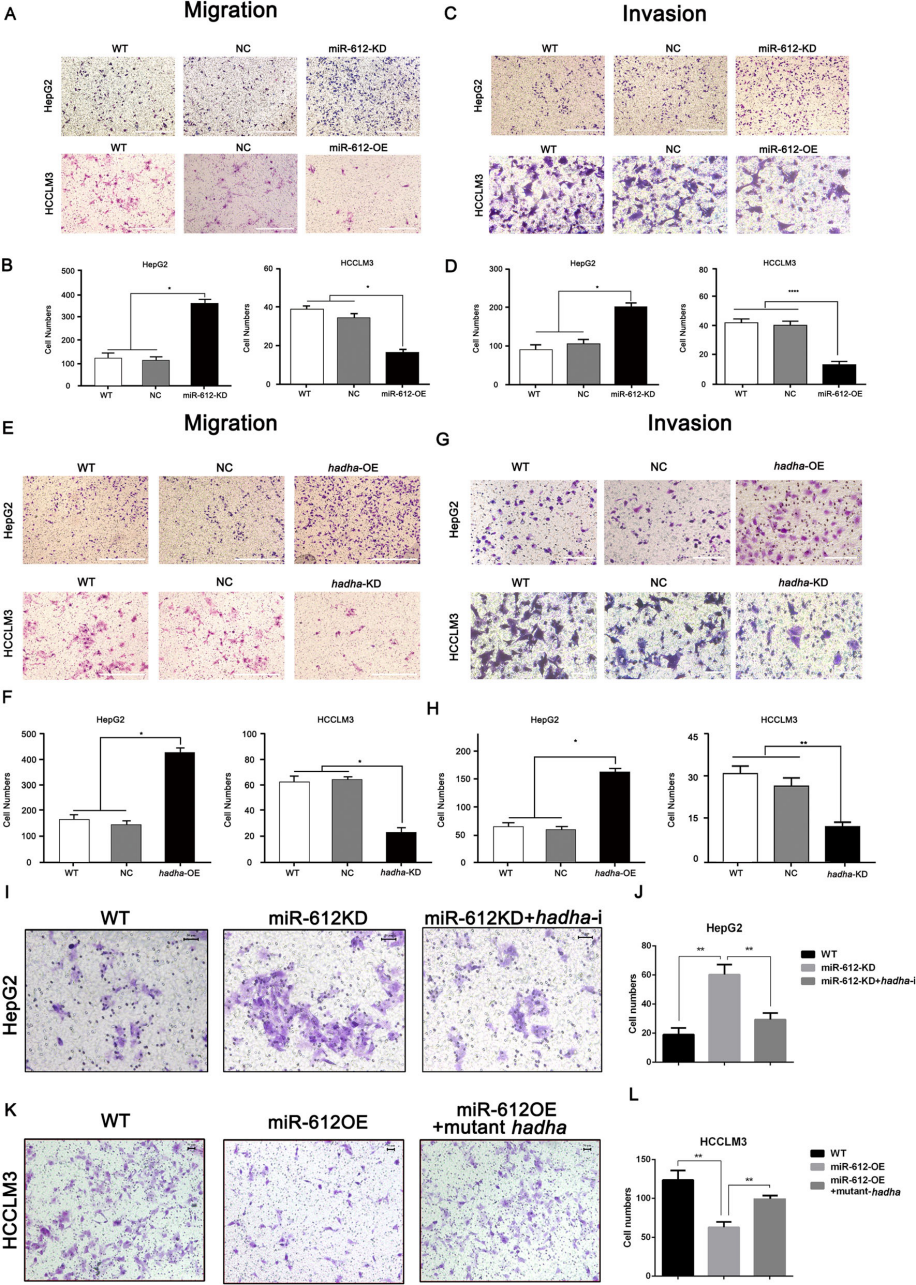
MiR-612 suppresses invasion and migration of HCC by targeting *hadha*. **a**, **b** Cell migration abilities and statistic results of HepG2^miR-612-KD^ and HCCLM3^miR-612-OE^ cells. Scale bars, 200 μm. **c**, **d** Cell invasion abilities and statistic results of HepG2^miR-612-KD^ and HCCLM3^miR-612-OE^ cells. Scale bars, 200 μm. **e**, **f** Cell migration abilities and statistic results of HepG2^*hadha*-OE^ and HCCLM3^*hadha*-KD^ cells. Scale bars, 200 μm. **g**, **h** Cell invasion abilities and statistic results of HepG2^*hadha*-OE^ and HCCLM3^*hadha*-KD^ cells. Cell migration abilities and statistic results of HepG2^miR-612-KD^ cells after *hadha* was knocked down. Scale bars, 200 μm. **i**, **j** Cell migration abilities and statistic results of HCCLM3 ^miR-612-OE^ cells after HADHA was rescued. Scale bars, 50 μm. **k**, **l** Cells (*p < 0.05; **p < 0.01). Data are mean ± SEM of three independent experiments. Scale bars, 50 μm
