# Supplementary figures and images for: MiR-612 regulates invadopodia of hepatocellular carcinoma by HADHA-mediated lipid reprogramming
Source: J Hematol Oncol. 2020 Feb 7;13:12. doi: 10.1186/s13045-019-0841-3 (PMC7006096; doi:10.1186/s13045-019-0841-3)

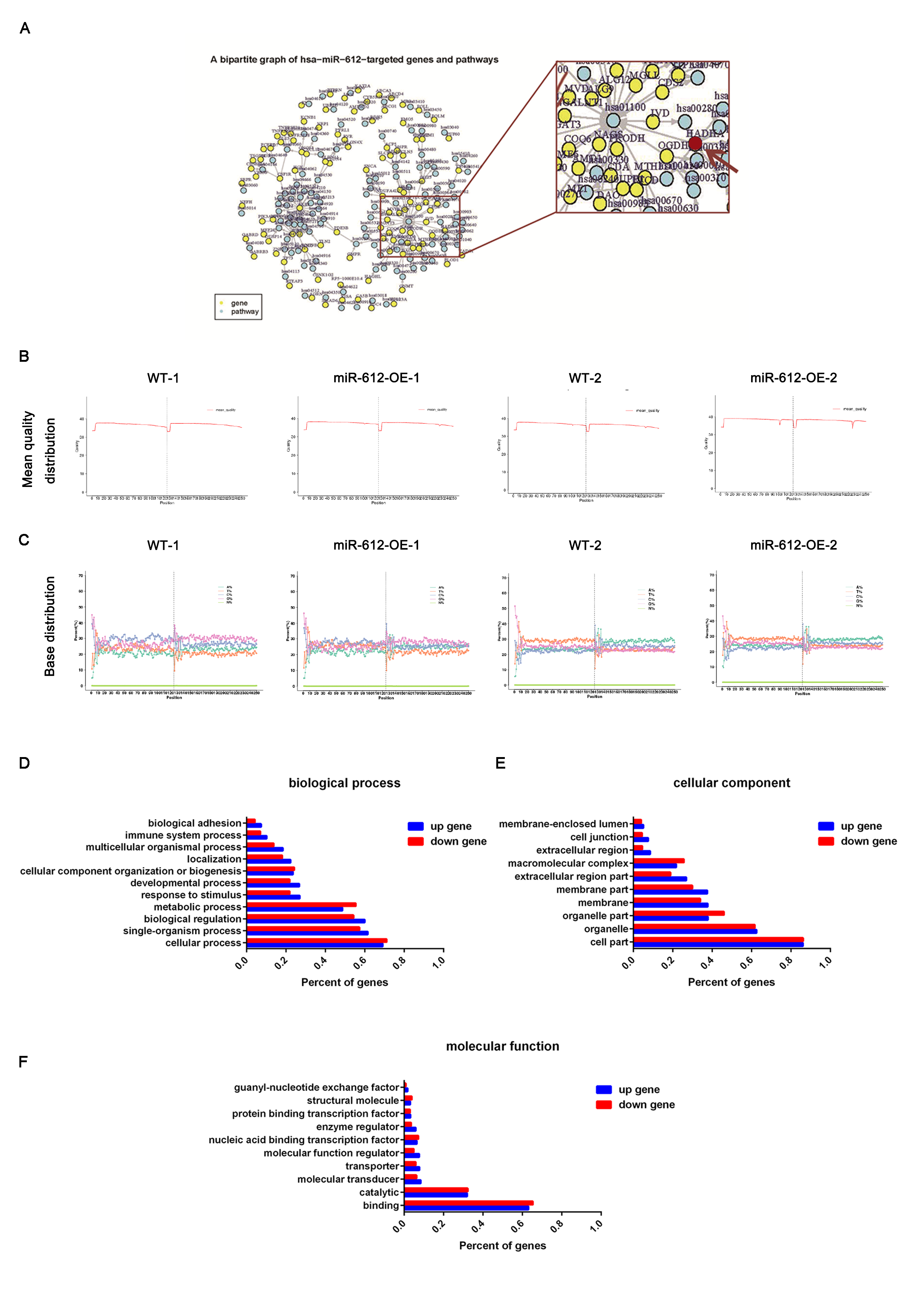

Supplement: Supplementary file 1 — Additional file 1:. Figure S1. (A) Target genes and signaling pathways of miR-612 predicted by bioinformatics analyses. Red spot stands for HADHA. (B) Quality distribution of RNA pulled down by biotin-labeled miR-612 for RNA-seq analyses. (C) Base distribution of RNA pulled down by biotin-labeled miR-612 for RNA-seq analyses. (D-F) GO analyses of differential down-regulated genes based on their molecular function, cellular component and biological process. [file 13045_2019_841_MOESM1_ESM.tif]

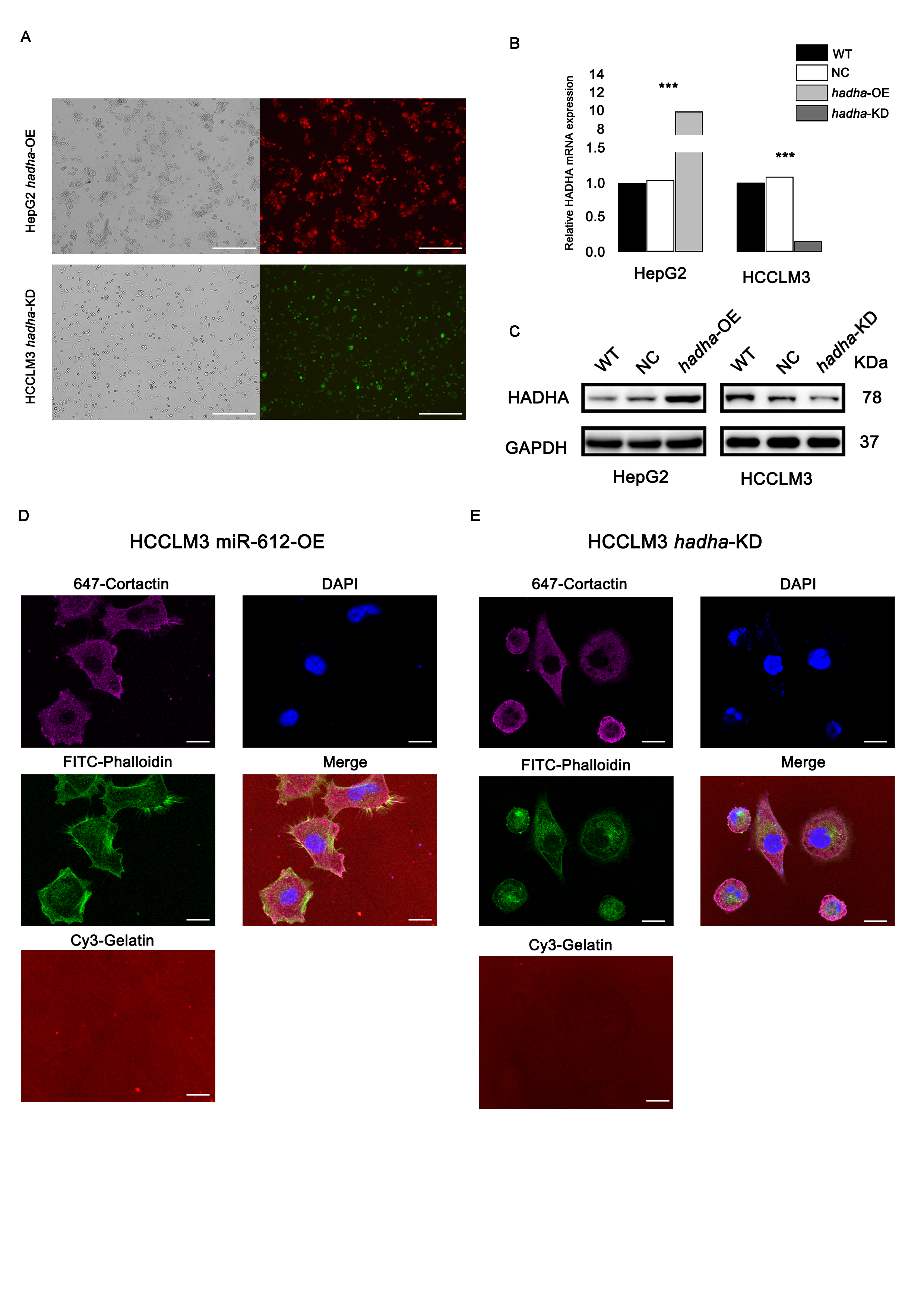

Supplement: Supplementary file 2 — Additional file 2: Figure S2. (A) HepG2 and HCCLM3 cells infected with hadha overexpression or knockdown lentivirus respectively. WT represents wild type cell line without any treatment. NC samples means the cell lines treated with negative control lentivirus. Scale bars, 200 μm. (B and C) The mRNA and protein levels of HADHA were tested by real-time PCRs and Western blots. (D and E) Fluorescence images of invadopodia staining by 647(Cortactin) and FITC (phalloidin) in HCCLM3miR-612-OE and HCCLM3hadha-KD cells. (***p < 0.001). Data are mean ± SEM of three independent experiments. Scale bars, 15 μm. [file 13045_2019_841_MOESM2_ESM.tif]

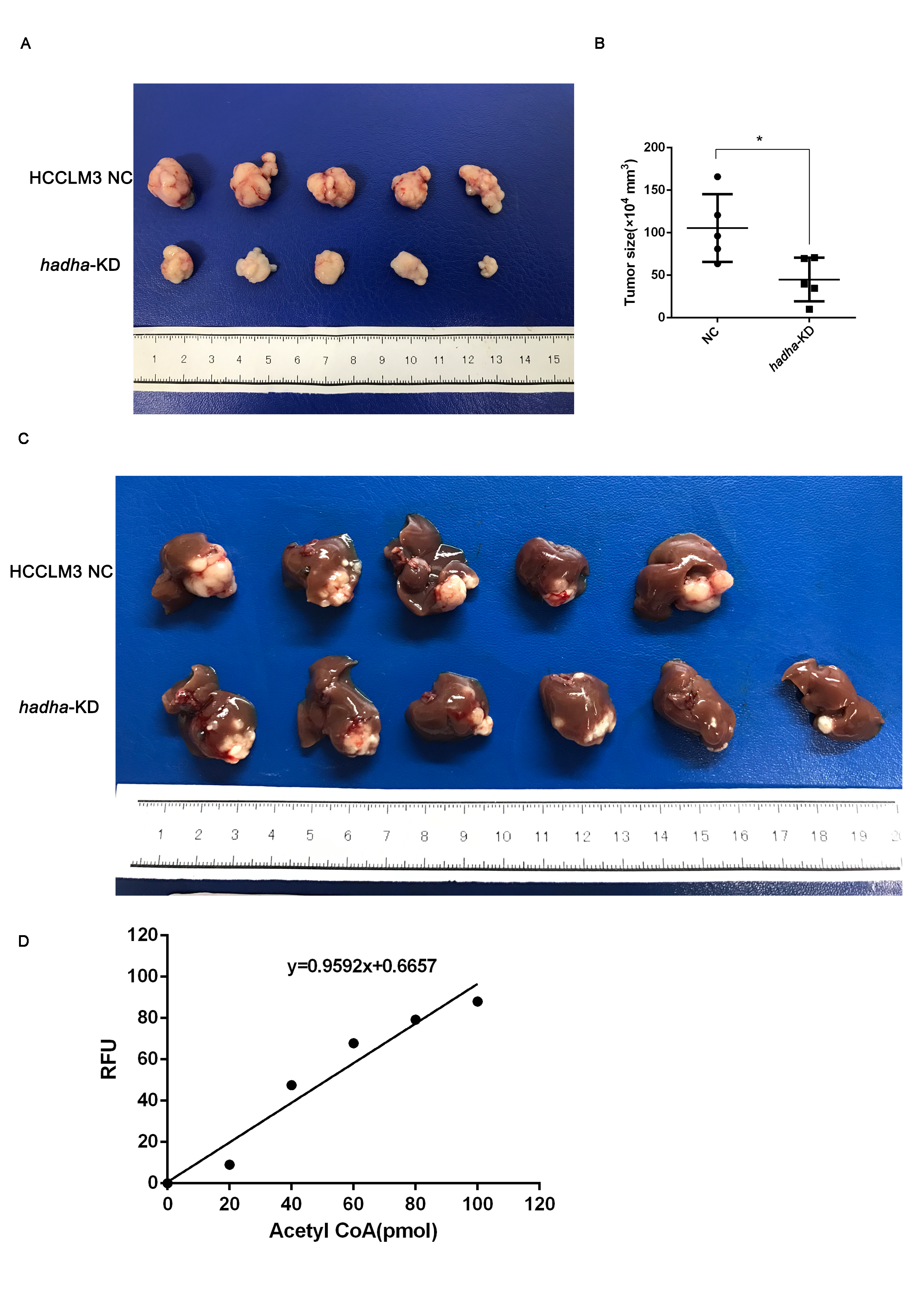

Supplement: Supplementary file 3 — Additional file 3: Figure S3. (A and B) Tumor sizes and statistic results of subcutaneous HCCLM3NC and HCCLM3hadha-KD xenografts. (C) Tumor sizes of HCCLM3NC and HCCLM3hadha-KD xenografts in liver. (D) Standard plots of Acetyl CoA (*p < 0.05). [file 13045_2019_841_MOESM3_ESM.tif]
