# Supplementary material for: MiR-612 regulates invadopodia of hepatocellular carcinoma by HADHA-mediated lipid reprogramming
Source: J Hematol Oncol. 2020 Feb 7;13:12. doi: 10.1186/s13045-019-0841-3 (PMC7006096; doi:10.1186/s13045-019-0841-3)
Supplement: Supplementary file 5 — Additional file 5: Table S1. Primers sequences of PCR. Table S2. Reaction Mixes for Acetyl-Coenzyme A Assay (unit: μl). Table S3. ATP detection operation rules (unit: μl). Table S4. Reaction mixes for cholesterol quantitation assay (unit: μl). [file 13045_2019_841_MOESM5_ESM.docx]

**Supplementary Table 1. Primers sequences of PCR**

| Gene | Sequence |
| --- | --- |
| has-miR-612 | 5’- GCAGGGCTTCTGAGCTCCTTAA -3’ |
| U6 small nuclear RNA (U6) | 5’- CAAATTCGTGAAGCGTTCCATAT -3 |
| HADHA(forward) | 5’-AGTAGAAGCGGTGATTCCAGA-3’ |
| HADHA(reverse) | 5’-CCACGGGAGAGAAGTAGTGC-3’ |

**Supplementary Table 2. Reaction Mixes for Acetyl-Coenzyme A Assay (unit: μl)**

| Reagent | Samples and Standards | Blank Sample |
| --- | --- | --- |
| Acetyl-CoA Assay Buffer | 41.8 | 42.8 |
| Acetyl-CoA Substrate Mix | 2 | 2 |
| Conversion Enzyme | 1 | - |
| Acetyl-CoA Enzyme Mix | 5 | 5 |
| Fluorescent Probe | 0.2 | 0.2 |

**Supplementary Table 3. ATP detection operation rules (unit: μl)**

|  | Blank tube | Standard tube | Measuring tube | Control tube |
| --- | --- | --- | --- | --- |
| 1mol/l standard solution | 30 | 30 | - | - |
| sample | - | - | 30 | 30 |
| Reagent 1: Substrate I | 100 | 100 | 100 | 100 |
| Reagent 2: Substrate II | 200 | 200 | 200 | 200 |
| Reagent 3: Accelerator | - | 30 | 30 | - |
| Double distilled water | 30 | - | - | 30 |
| Mix well, 37 ° C water bath for 30 minutes | | | | |
| Reagent 4: Precipitant | 50 | 50 | 50 | 50 |
| After thoroughly mixing, centrifuge at 4000 rpm for 5 minutes,  and take 300 μl of the supernatant for measurement. | | | | |
| Sample supernatant | 300 | 300 | 300 | 300 |
| Reagent 5: coloring solution | 500 | 500 | 500 | 500 |
| Mix and place for 2 minutes at room temperature | | | | |
| Reagent 6: Terminator | 500 | 500 | 500 | 500 |

**Supplementary Table 4. Reaction mixes for cholesterol quantitation assay (unit: μl)**

| Component | Samples well | Standard well |
| --- | --- | --- |
| Samples + Cholesterol Assay Buffer | 10 + 40 | - |
| Standard dilutions | - | 50 |
| Cholesterol Assay Buffer | 44 | 44 |
| Cholesterol Probe | 2 | 2 |
| Cholesterol Enzyme Mix | 2 | 2 |
| Cholesterol Esterase | 2 | 2 |
