# Supplementary material for: MiR-612 regulates invadopodia of hepatocellular carcinoma by HADHA-mediated lipid reprogramming
Source: J Hematol Oncol. 2020 Feb 7;13:12. doi: 10.1186/s13045-019-0841-3 (PMC7006096; doi:10.1186/s13045-019-0841-3)
Supplement: Supplementary file 6 — Additional file 6. Other materials and methods. [file 13045_2019_841_MOESM6_ESM.docx]

**Other materials and methods**

**Protein levels detected by Western blot analysis**

Lysates were obtained from cultured cells with a mixture of ProteoJET Mammalian Cell Lysis Reagent (Thermo Fisher Scientific, MA, USA) and PMSF (Roche, Basel, Switzerland). About 20μg protein was extracted from each sample, separated by 10% SDS-PAGE and transferred onto polyvinylidene fluoride membranes. After being blocked in 5% bovine serum albumin, the interested protein was probed with antibodies against human HADHA (1:1000; Abcam, MA, USA), Cortactin (1:1000; Cell Signaling Technology, MA, USA), Caveolin-1 (1:1000; Cell Signaling Technology), E-cadherin (1:1000; Cell Signaling Technology), β-catenin (1:1000; Cell Signaling Technology), MMP14 (1:500; Proteintech), Slug (1:1000, Cell Signaling Technology), Vimentin (1:1000, Cell Signaling Technology), GAPDH (1:3000, Abcam), and incubated with goat antirabbit or anti-mouse IgG (1:10000 for both; Jackson ImmunoResearch Laboratories, PA, USA), and detected with enhanced chemiluminescence reagents (Thermo Fisher Scientific). The bands were visualized using 1-stepTM NBT/BCIP reagents (Thermo Fisher Scientific) and detected by Tanon 5200 automatic chemiluminescence image analysis system (Tanon, Shanghai, China).

**RNA extraction and real-time PCR assays for mRNA detection**

Total RNA was extracted from cultured cells with Trizol Reagent (Thermo Fisher Scientific). The quality and integrity of RNA were evaluated via A260/A280 ratio, and then 1μg of total RNA was used for first-strand DNA synthesis. Real-time PCR was performed in triplicate by the SYBR Green PCR method using an All-in-One miRNA qPCR Detection kit (GeneCopoeia, MD, USA). The forward primers of has-miR-612 and U6 small nuclear RNA (U6) were synthesized as (Supplementary Table 1)[24]. The common reverse primer was purchased from the same company. For mRNA detection, 1μg of total RNA was used for complementary DNA synthesis with a PrimeScript RT reagent kit (Takara Bio, Kyoto, Japan). Real-time PCR was performed in triplicate using SYBR Premix Ex Taq (Takara Bio). The primers for *hadha* were synthesized by Sangon Biotech (Sangon Biotech, Ltd, Shanghai, China) as Supplementary Table1. The *U6* and *gapdh* were used as internal control for miRNAs and mRNAs assays, respectively. The threshold cycle (Ct) values were analyzed using the comparative Ct (−ΔCt) method[26]. The level of targets was obtained by normalizing to the endogenous reference and relative to a control.

**RNA immunoprecipitation and sequencing**

MiR-612 was labelled with biotin (Sangon Biotech, Ltd, Shanghai, China) and specifically bonded to streptomycin pre-labelled magnetic beads (Thermo Fisher Scientific). MiR-612-interacted RNAs were pulled down by two independent RNA immunoprecipitation assays and then sequenced (Annoroad Genome, Beijing, China) (Supplementary Fig.1B, C) [27]. DEseq was used to analyze their differential expressed levels. Targets were available when |log2Ratio|≥1 and q<0.05, and listed in Supplementary Excel1.

**Luciferase reporter assay**

The binding sites for miR-612 in the 3’-UTR sequence of *hadha* were cloned into the pMIR, (Promega, WI, USA). These constructs with mutant and blank plasmids were co-transfected into target cells in 96-well plates together with miR-612 mimics using Lipofectamine 3000 (Thermo Fisher Scientific). Luciferase activity was measured 24h after transfection using the Dual-Luciferase Reporter Assay System (Promega). The levels of firefly luciferase activities were obtained by normalizing to Renilla luciferase activities and relative to a control, as previously reported[28].

**MiRNA in situ hybridization**

TMA slides were incubated at 60°C for 1h, deparaffinized in xylene, and rehydrated with graded alcohol solutions. Slides were then washed three times with RNase-free PBS, digested with 8 mg/ml pepsin at 3°C for 10mins, washed, and then dehydrated in graded alcohol solutions. Slides were hybridized at 40°C overnight with 50 nm locked nucleic acid (LNA)-modified DIG-labeled probes against miR-612 (Qiagen, Duesseldorf, Germany). After stringency washes (5×, 1×, 0.2× SSC), slides then were placed in blocking buffer for 30mins at room temperature followed by overnight incubation at 4°C in alkaline phosphatase conjugated anti-DIG Fab fragment. Antibody signals were colorized with NBT and BCIP substrate (Roche, Mannheim, Germany) and then nuclei were stained by Nuclear Fast Red.

**Patient selection and TMA construction**

Patients who meet the following criteria were eligible for the study between July 2011 and July 2014. At the age of 18-70 years old and signed written informed consent. Patients with complete laboratory test results were underwent curative liver resection in Zhongshan Hospital (Shanghai, China), and the [pathological](javascript:;) [diagnosis](javascript:;) should be [primary](javascript:;) [hepatocellular](javascript:;) [carcinoma](javascript:;). Patients combined with other tumors，or with a history of immunodeficiency, including HIV positive or other acquired or congenital immunodeficiency disorders, or a history of organ transplantation were not eligible for the study. One hundred and thirty-four patients were recruited in the study. The follow-up information was updated until 1 May 2018.

All HCC tissues and adjacent normal liver tissues were obtained and made into TMA according to previous method [29]. All procedures were approved by the Zhongshan Hospital Research Ethics Committee. Informed consent was obtained from each patient according to regulations set forth by Ethics Committee.

[**Immunofluorescence**](javascript:void(0);) **staining**

HCC cells were re-suspended in fresh medium, and adjusted to a concentration of 1×10^4^ cells/10μl into a 24-well glass dish. Monolayer cells grew into 70%-80% confluency, and then were fixed with 4% paraformaldehyde. Next, the cells were incubated with Cortactin antibody (1: 250) and Caveolin-1 antibody (1: 200) in 4℃overnight followed by Alexa Fluor 488-donkey anti-rabbit IgG (1: 250; ProteinTech Group, Hubei, China) and Alexa Fluor 594-conjugated goat anti-mouse IgG (1: 250; ProteinTech Group) for 1h in the dark. The cell nuclei were counterstained with DAPI (Sangon Biotech, Ltd). Images were obtained under the Laser Scanning Confocal Microscope-TCS SP5 (Leica, Heidelberg, Germany).

**Immunohistochemical staining**

Immunohistochemistry staining of target protein was performed on TMA. The slides were probed with a primary antibody against HADHA (1:1000), Cortactin (1:1000), Caveolin-1 (1:1000), and then incubated with horseradish peroxidase-conjugated IgG (1: 500; Thermo Fisher Scientific), and the proteins in situ were visualized with 3, 3’-diaminobenzidine. The intensity of positive staining was measured with integrated optical density (IOD) twice independently. In detail, the density of all stained chip were measured by a computerized image system, Leica DM IRE2 microscope(Leica Microsystems Imaging Solutions Ltd, Cambridge, United Kingdom) and photographed under 200 magnified visual field in the same parameters. Image-proplus v6.0 software(Media Cybernetics Inc, Bethesda, MD) was used for analysis. The typical tan color was used as the positive staining of target protein. IOD value was calculated according to the positive staining intensity and area. Staining intensity of target protein also named mean IOD (mIOD) was acquired through dividing by the area of tissue.

**In vitro migration and invasion assays**

Cell migration and invasion were analyzed by a Transwell Permeable Supports system with 8-µm pores (Corning，New York, USA). For motility assays, 5×10^4^ cells were seeded into upper uncoated inserts; for invasion assays, 1.0×10^5^ cells were seeded into upper inserts with a Matrigel-coated membrane (BD, New Jersey, USA). Cells were seeded in 1% serum medium and translocated to 10% serum media for 24h or 48h. After removal of the nonmigrating or non-invading cells, the remaining cells were fixed, stained, and analyzed by inverted microscopy (Olympus, Tokyo, Japan)[19].

**Wound healing assays**

When cells grew into 90% of confluency, a scratch wound in the monolayer was made using a pipette tip. After washing away all detached cells with PBS, and then the distances of wounds were measured by microscope at 0, 24h and 48h after treatment[31].

**In vivo assays for tumor metastasis**

Male athymic BALB/c nude mice of 4-6 weeks old (Vital River Laboratories, Beijing, China) were used for animal studies. An orthotopic human HCC xenograft model was established for in-vivo tumor invasion and metastasis analysis[32]. The cell suspension with 6.0×10^6^/200μl (per mouse) was injected subcutaneously into the shoulder back of male BALB/c nude mice. After 4 weeks of culture in SPF laminar flow room, the mice were sacrificed and their tumors were collected in the sterile ultra-clean table for use. Tumor sizes were evaluated by the formula: Volume (mm3) = [width^2^ (mm^2^) × length (mm)]/2. Similar tissue sizes (2*2*2mm^3^) of subcutaneous xenograft of HCCLM3*^hadha^*^-KD^ and HCCLM3^NC^ were transplanted into mouse livers. All mice were monitored once every 5 days and living metastasis foci in lung were evaluated by Quantum GX micro-CT Imaging System (PerkinElmer, MA, USA) 6 weeks later. After sacrificed, livers and lungs were removed and all the metastatic nodules were megascopic and eyes for counting the nodule number. Then they were embedded in paraffin and total metastatic foci in HE staining were counted under microscope, as described previously[33]. Cortactin and Caveolin-1 were stained by immunohistochemistry. All procedures were approved by the Animal Care and Use Committee of Shanghai, China.

**Cell membrane fluidity evaluated by TMA-DPH**

Membrane fluidity was measured by TMA-DPH fluorescent probes according to manufactures protocol. In belief, 50mM storaged probes against TMA-DPH (No. 2149, AAT Bioquest, Hubei, China) were firstly diluted into 5μM working probes with DMSO, and adjusted pH value with Hanks’ buffer (20mM Hepes, pH7.4). Then 90% confluency of HCC cells with indicated treatment in 96-well plate were incubated with 200μl working probes against TMA-DPH in the dark at 37℃, 5% CO_2_ incubator for 5mins. After washed twice, the cells were re-suspended in Hanks’ buffer (20mM Hepes, pH 7.4) and the fluorescence intensity was measured by fluorescence spectrophotometer at λex=360nm/λem=460nm. Fluorescence Polarization (FP)=1000×(IVV-GIVH)/(IVV+GIVH), in which IVV was fluorescence intensity of the polarizer and the optical axis both in vertical, IVH was fluorescence intensity of the polarizer in vertical and the optical axis in horizontal and G—correction factor.

**Cell lipid extraction for (LC-MS)/MS detection**

About 0.9 mL water was added into cell pellet(scraped from 60 mm cell culture dish) in 15 mL tube and vertex for 10s, and the cell structure was disrupted under ultrasonic condition for 30 seconds. After that, freezing and thawing was applied for at least three cycles. Secondly, we added 1 ml CHCl3 and 2 mL MeOH to above treated sample and vortex for 30 seconds. Next, 1ml CHCl3 and 1ml water was added in order, and centrifuge for 10 minutes at 3200rpm. Then, we pipetted the bottom organic layer to a new tube for air drying and re-constituted the residues in 200 μL chloroform:methanol (1:2, v/v, including 10mM NH4Ac). Finally, we pipetted 2μl into the specific glass bottle for detecting.
